# Multiple-pathways light modulation in *Pleurosigma strigosum* bi-raphid diatom

**DOI:** 10.1038/s41598-024-56206-y

**Published:** 2024-03-18

**Authors:** Edoardo De Tommasi, Ilaria Rea, Maria Antonietta Ferrara, Luca De Stefano, Mario De Stefano, Adil Y. Al-Handal, Marija Stamenković, Angela Wulff

**Affiliations:** 1 National Research Council, Institute of Applied Sciences and Intelligent Systems “E. Caianiello”, Unit of Naples, Via P. Castellino 111, 80131 Naples, Italy; 2 Department of Environmental, Biological, and Pharmaceutical Sciences and Technologies, University of Campania “Luigi Vanvitelli”, Via Vivaldi 43, 81100 Caserta, Italy; 3 Department of Biological and Environmental Sciences, University of Gothenburg, Box 463, 405 30 Göteborg, Sweden; 4 Department of Ecology, Institute for Biological Research “Sinisa Stankovic”, University of Belgrade, Bulevar despota Stefana 142, Belgrade, 11060 Serbia

**Keywords:** Photonics in nature, Biophotonics, Nanostructured biomaterials, Diatoms, Optics and photonics, Biophotonics

## Abstract

Ordered, quasi-ordered, and even disordered nanostructures can be identified as constituent components of several protists, plants and animals, making possible an efficient manipulation of light for intra- and inter- species communication, camouflage, or for the enhancement of primary production. Diatoms are ubiquitous unicellular microalgae inhabiting all the aquatic environments on Earth. They developed, through tens of millions of years of evolution, ultrastructured silica cell walls, the frustules, able to handle optical radiation through multiple diffractive, refractive, and wave-guiding processes, possibly at the basis of their high photosynthetic efficiency. In this study, we employed a range of imaging, spectroscopic and numerical techniques (including transmission imaging, digital holography, photoluminescence spectroscopy, and numerical simulations based on wide-angle beam propagation method) to identify and describe different mechanisms by which *Pleurosigma strigosum* frustules can modulate optical radiation of different spectral content. Finally, we correlated the optical response of the frustule to the interaction with light in living, individual cells within their aquatic environment following various irradiation treatments. The obtained results demonstrate the favorable transmission of photosynthetic active radiation inside the cell compared to potentially detrimental ultraviolet radiation.

## Introduction

The capacity to manipulate light has undergone significant development in recent decades, with applications spanning telecommunications, imaging, energy harvesting, medicine, and extending to more cutting-edge domains like quantum computing and cryptography^1^. The discovery of new materials and advancements in fabrication techniques have enabled the handling of light at the micro- and nano-scale^2^. The introduction of metallic and dielectric metasurfaces has further empowered precise control over the intensity, phase, and polarization of incident light, offering functionalities traditionally achieved through combinations of conventional optical components^3,4^.

Despite these remarkable achievements, nature, shaped through billions of years of evolution, has anticipated human endeavors in various aspects. Several protists, plants, and animal species exhibit ordered, quasi-ordered, or disordered structures at the sub-micron scale capable of interacting with optical radiation in more or less intricate ways^5^. A number of beetles, butterflies, and even birds leverage multilayer interference to reflect optical radiation within specific spectral ranges, resulting in structural colors that serve purposes in intra- and inter-species signaling and camouflage^6,7^ More complex effects include helicity-sensitive reflection of circular polarized light^8^, and high levels of whiteness^9^, blackness^10^ or wide-angle transparency^11^ ascribed to disordered nanostructures. Beyond the animal kingdom, multilayer reflectors and diffraction gratings are also found in plant leaves and flower petals, enhancing photosynthetic efficiency^12^ and facilitating the attraction of pollinators^13^.

Although diatoms likely originated over 200 million years ago^14^, their optical properties exhibit similarities to recent artificial photonic devices^15^. Diatoms, ubiquitous unicellular microalgae, have colonized all aquatic environments, contributing significantly to global primary production (20–25%^16^) and annual silica precipitation (about 240 Tmol^17^). Enclosed in a hydrogenated porous silica cell wall called frustule, diatom cells undergo a finely-tuned biomineralization process, the genetic control of which remains incompletely understood^18^. The frustule, ranging from a few micrometers to about 1 millimeter in linear dimensions, consists of an epitheca overlapped to a hypotheca, each comprising a valve and one or more lateral bands (girdles). Valves and girdles are adorned with ordered or quasi-ordered patterns of pores, with diameters ranging from tens of nanometers to about one micron, depending on the location within the shell and the species.

The taxonomy of diatoms is based on frustule shape, although the genetic species concept is advancing, resulting in the identification of approximately $10^3$ genera and an estimated $10^5$ species, establishing diatoms as one of the most diverse groups of eukaryotes^19^. The frustule is not merely a structural marvel; its morphological elegance is intricately linked to its functionality. Pores and ridges reduce mechanical stress, allowing the frustule to withstand pressures ranging from 1 to 7 N/mm^2^, equivalent to 100–700 t/m^2^^20^. Furthermore, the pores connect the diatom cell with its environment, facilitating nutrient and silicic acid sorting, protection against harmful agents (e.g., viruses and bacteria), enabling gas exchange and the release of waste products^21^. Additionally, the regular distribution of pores, with dimensions comparable to visible radiation wavelengths, enhances the effective coupling of frustules with light, potentially explaining diatoms’ impressive photosynthetic efficiency^22^.

Optical properties observed through years in centric (i.e. radially symmetric) diatom frustules include photonic crystal-like behavior of *Coscinodiscus granii* girdles^23,24^ and *Melosira variance* girdles and valves^25^; diffraction-induced optical lensing in *Coscinodiscus wailesii*^26^, *Arachnoidiscus* sp.^27^, *Coscinodiscus centralis*^28,29^, and *C. granii*^30^ valves; manipulation of the polarization state of the incoming radiation (optical activity) in *Aulacodiscus oregonus*^31^ and *Arachnoidiscus ehrenbergii*^32^ valves; frustule photoluminescence in *Thalassiosira rotula*^33^, *Coscinodiscus concinnus*^34^, *C. wailesii*^35^ and several other species^36,37^; ultraviolet radiation (UVR) selective screening by *C. wailesii*^35^ and *Coscinodiscus radiatus*^38^ valves.

Recently, research on the optical properties of diatoms has expanded to include the evolutionarily younger pennate diatoms, characterized by bilaterally symmetric frustules and primarily benthic^39^. A comparative study with centric diatoms regarding their interaction with UVR is reported in Aguirre et al.^38^. More recently, D’Mello et al.^40^ employed finite-difference time-domain (FDTD) numerical simulations and scanning near-field optical microscopy (SNOM) to study the optical behavior of the individual components of a *Nitzschia filiformis* frustule, including valves, girdle, raphe, and apices. This research also explored how spatial disorder in the location of pores influences light coupling efficiency. Ghobara et al.^41^ utilized the finite element method (FEM) in the frequency domain to investigate near-field light modulation by the *Gomphonema parvulum* frustule, extracting its overall response by superimposing different optical phenomena associated with its individual components. While these works demonstrated high accuracy, they were confined to bare frustules. The main difficulty, in this kind of studies, is to relate the photonic properties of an inorganic, ultrastructured material, and the behavior and performances of a living organism incapsulated in it. An effort to characterize the optical features of a live pennate diatom in its aquatic environment was accomplished in our recent study on *Ctenophora pulchella* araphid (non-motile) diatom, where it is shown how the frustule efficiently collects photosynthetic active radiation (PAR, $\lambda =400-700$ nm), confining it in light patterns spatially superimposed to plastids, while simultaneously attenuating potentially detrimental UVR^42^.

**Figure 1:**
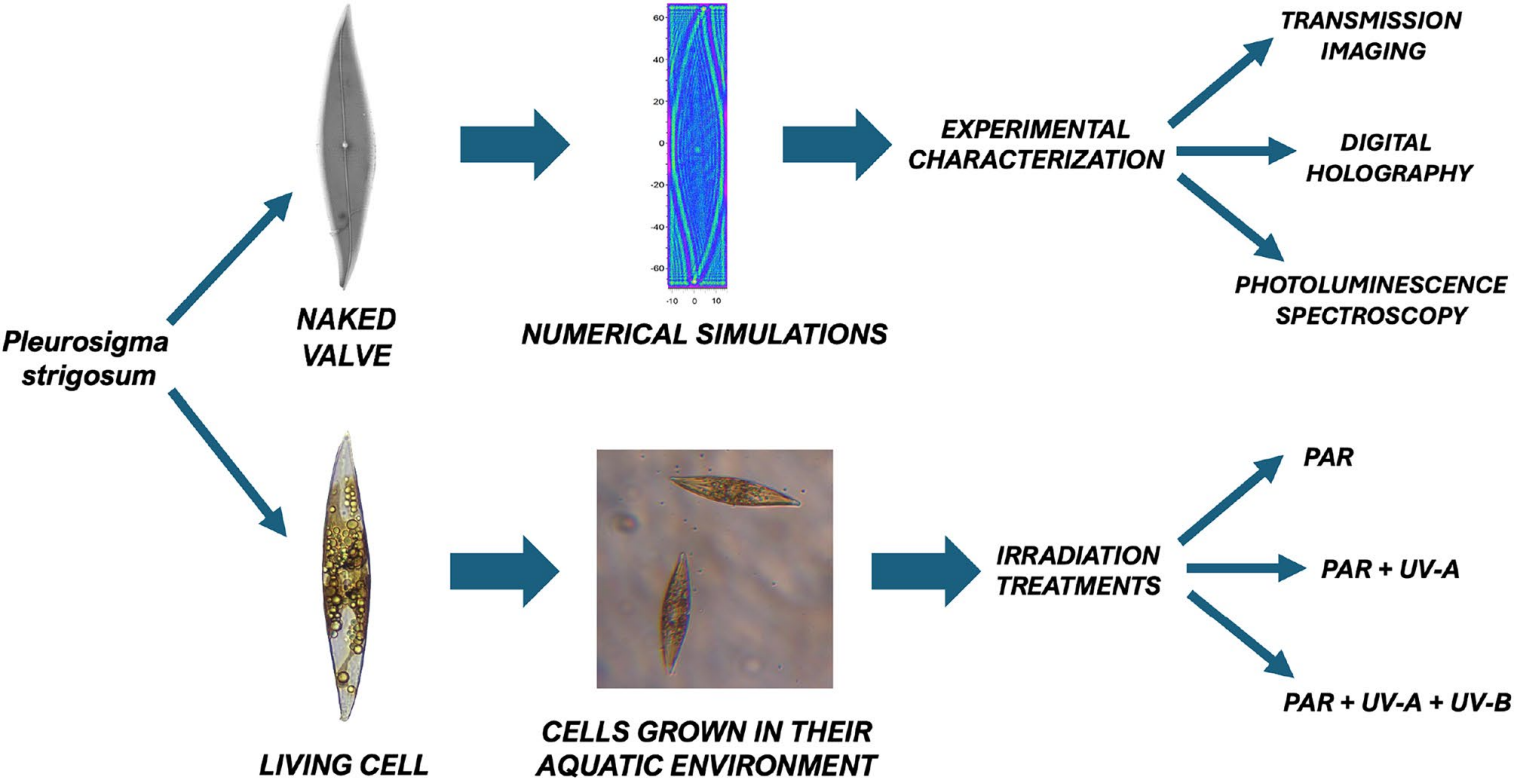
Conceptual scheme of the applied methodology used to study the photonic properties of *Pleurosigma strigosum*. Following the removal of organic content from the cell, individual valves are extracted and morphologically characterized using scanning electron microscopy (SEM). SEM characterization facilitates the retrieval of accurate CAD models for simulating light propagation through single valves in different spectral ranges. Numerical results are then compared with transmission imaging (for both PAR and UVR). The optical characterization of the valve is further complemented by digital holography and photoluminescence spectroscopy. Simultaneously, living cells are cultivated in their aquatic medium and subjected to various irradiation treatments, including UVR. The obtained results, encompassing growth rates and photosynthetic parameters, are analyzed, taking into consideration the optical properties of the valve. This analysis aims to highlight the potential role of the valve in photosynthesis and photoprotection within the living organism.

Building on this investigative path, the present work analyzes how PAR and UVR interact with both bare frustules and live individual cells of the bi-raphid *Pleurosigma strigosum* diatom in their aquatic environment. In contrast to *C. pulchella*, cells of this pennate, raphid species are motile and possess the ability to change their orientation based on illumination conditions. Their optical response is strongly correlated with the spectral content of incoming radiation, resulting from a composite interaction of diffractive, refractive, scattering, guiding, and frequency down-conversion effects. By combining numerical simulations, multiple microscopy techniques, transmission imaging, digital holography, photoluminescence spectroscopy, and in vivo experiments on cell cultures subjected to different irradiation treatments, we have identified the primary mechanisms through which *P. strigosum* frustules efficiently collect PAR while simultaneously safeguarding the protoplast against potentially harmful, high-energy optical radiation. A scheme summarizing the methodologies used in this survey and their mutual relationships is reported in Fig. 1.

## Results and discussion

### *Pleurosigma strigosum*: main features and anatomy of the frustule

Diatoms belonging to the *Pleurosigma* genus are solitary and typically inhabit sandy or silty sediments as epipelon or epipsammon. However, some planktonic species have also been identified^43^, often as a result of resuspension. While they generally inhabit marine and brackish waters, they are occasionally found in high-conductivity freshwaters as well^44^.

**Figure 2:**
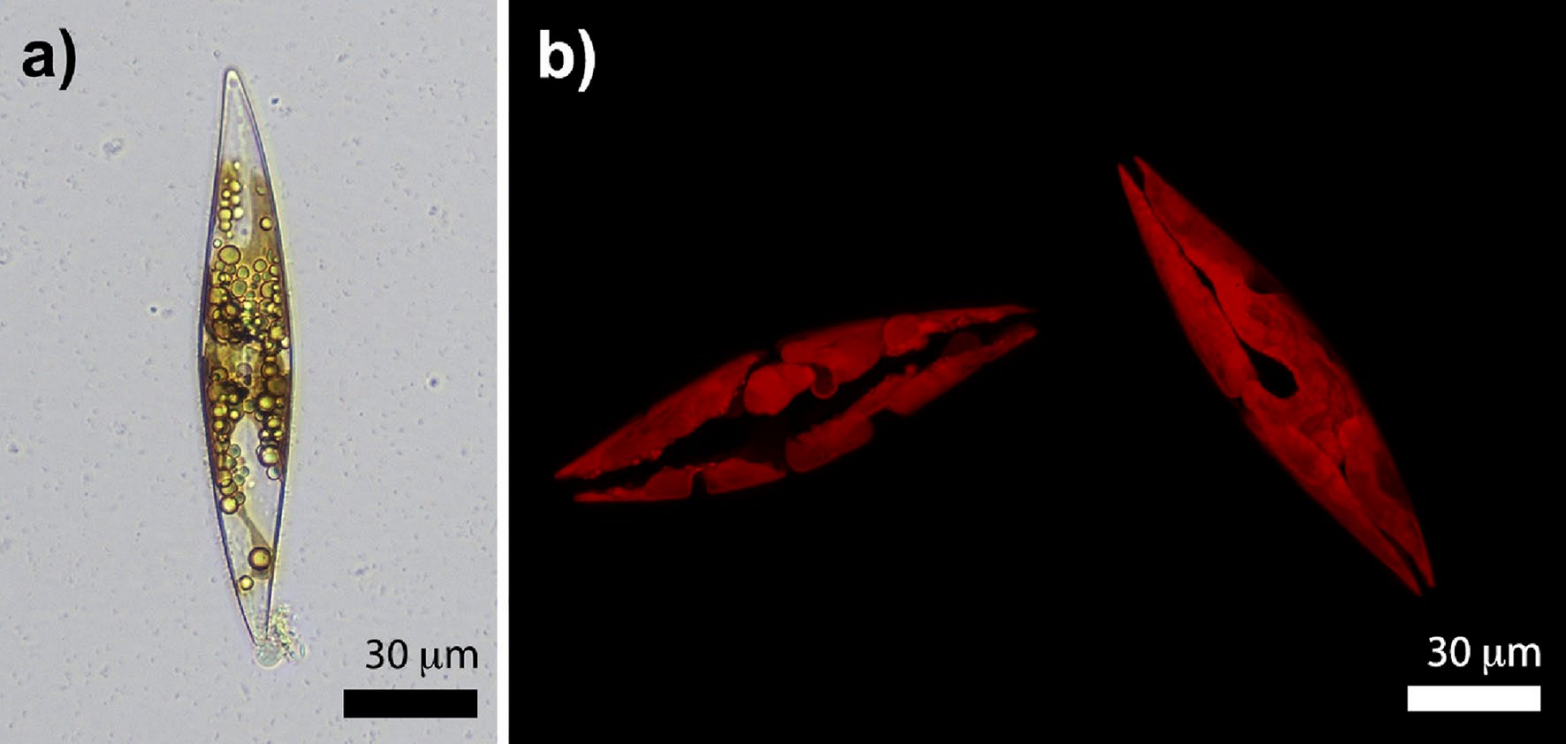
Optical micrograph of a single *P. strigosum* cell (**a**) and examples of 3D plastids reconstruction obtained by confocal laser scanning microscopy (**b**). Plastids autofluorescence is peaked at about $\lambda = 680$ nm following excitation by a diode laser emitting at $\lambda = 637$ nm.

Like all species in this genus, *P. strigosum* cells appear sigmoid in valvar view and are provided with two or four ribbon-like, irregularly-shaped plastids (one or two per theca). In Fig. 2a, spherical lipid droplets superimposed onto a plastid can be distinguished, with the plastid appearing brown due to the presence of the fucoxanthin pigment^45^. Plastid shape, volume, and location inside the cell can be more effectively discerned using confocal laser scanning microscopy (CLSM), enabling the three-dimensional reconstruction of plastids by exploiting chlorophyll autofluorescence emission in the red spectral range^26,46,47^ (Fig. 2b).

*P. strigosum* cells exhibit active movement and can rapidly change their orientation in response to variations in light exposure or other environmental changes. This motile mechanism is induced by the secretion of polymeric material from the raphe (composed of two slits along the valve), followed by its subsequent recollection at a central nodule^31,44^.

**Figure 3:**
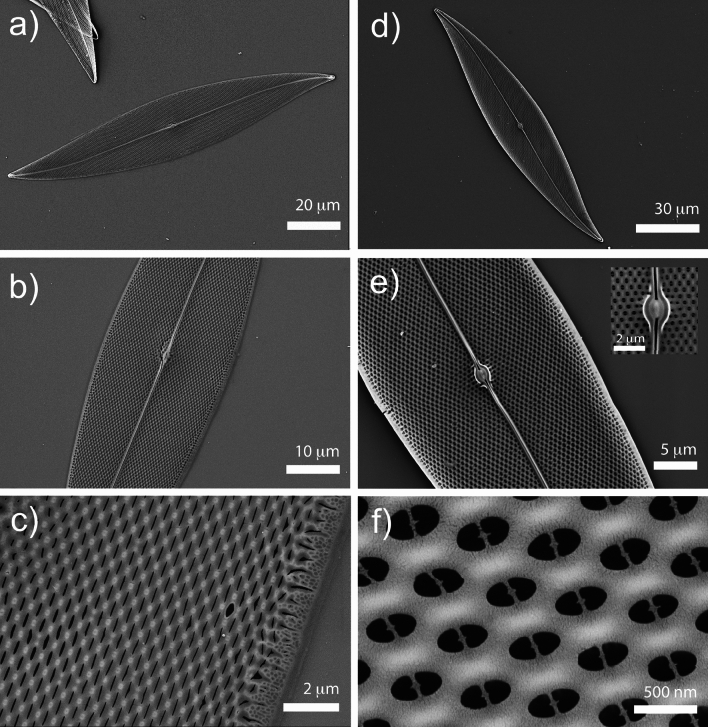
SEM images of a *P. strigosum* single valve at different magnifications. Outer (**a**–**c**, left column) and inner (**d**–**f**, right column) layer. Inset in (**e**): central oval nodule.

*P. strigosum* valves consist of an outer and an inner layer (see Fig. 3) separated by round or slightly oval pillars, resulting in a sandwich-type ultrastructure with an unobstructed interior, where all internal parts of the valve are in communication with each other^48^. This unique architecture ensures notable structural integrity against mechanical stress, such as torsion and shearing, with minimal silica consumption. In our strains, considering different growth stages, valves measured 115–254 µm in length and 25–34 µm in width, with a wall thickness of 200–400 nm. Valves exhibit a moderately to slightly sigmoid shape, narrowly lanceolate in longer specimens and somewhat broader in shorter ones, with acutely rounded apices. The areolae open to the outside through extremely narrow longitudinal slits, approximately 30 nm wide, and exhibit a lateral periodicity of about 600 nm (see Fig. 3c), while the inner layer is perforated by a hexagonal pattern of circular or oval pores, generally bisected by a narrow bridge, with an average diameter and lattice constant of about 300 nm and 600 nm, respectively (see Fig. 3f). The raphe sternum is characterized by a single curvature and by a raphe angle comprised between +7 and +10 degrees^49^. It looks almost straight for most of its length, gently curving near the apices, and it has an average width comparable to the lattice constant of the hexagonal pattern of pores (see Fig. 3e). The internal raphe endings converge towards a central, oval nodule flanked on either side by curved ridges (see the inset in Fig. 3e). The girdle contains open, non-porous bands. Considering mantle and girdle heights (1.5–3 µm and 10–14 µm, respectively), the height of the entire frustule measures about 13–20 µm.

### *P. strigosum* optical properties at first glance: a combined microscopy approach

**Figure 4:**
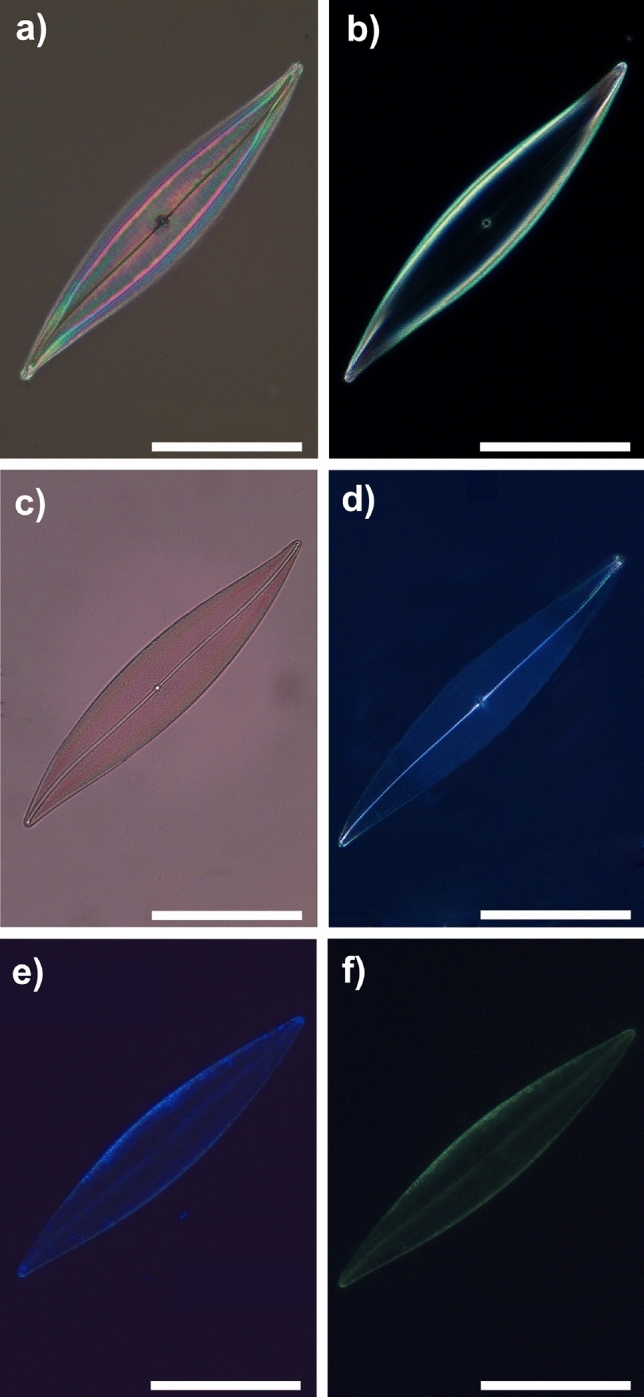
Bright field (**a**), dark field (**b**), transmission (**c**), cross-polarization (**d**), and fluorescence (**e**, **f**) images of a single *P. strigosum* valve. Scale bar: 50 µm. Excitation wavelengths employed in fluorescence imaging: 365 nm in (**e**) and 470 nm in (**f**).

Preliminary, qualitative information about the optical properties of a single *P. strigosum* valve can be easily obtained by examining images acquired through various microscopy techniques. When illuminated by a broadband (halogen) lamp and observed in bright field (see Fig. 4a), the valve exhibits strong iridescence due to diffraction induced by pores, whose linear dimensions are comparable to the wavelength of visible light. Owing to the periodicity of the pores, the valve functions indeed as a micro-grating, deviating different wavelengths along different directions. Dark-field microscopy enables imaging of the far-field optical response of the valve, identifying regions that contribute mainly to light scattering, i.e., the valve edges and the central nodule ridges (Fig. 4b). The nodule, along with the valve apices, also generates light focusing through a lensing effect, as observed in valve transmission (see Fig. 4c), analogous to the refraction induced by the *Ctenophora pulchella* central fascia^42^. Crossed-polarization imaging (Fig. 4d) eliminates transmitted radiation, enabling the detection of partially depolarized light coupled with the sternum. This suggests the presence of a waveguiding effect along the sternum itself. When irradiated by a LED source at 365 nm in the ultraviolet A region (UV-A) ($\lambda =315-400$ nm), blue light emission is detectable (Fig. 4e), transitioning to green when the valve is excited at 470 nm (Fig. 4f).

This initial, straightforward microscopy characterization already provides insights into how light manipulation by the *P. strigosum* valve occurs through multiple pathways, involving light diffraction, scattering, refraction, and optical confinement, depending on the region of the valve under observation. A further contribution to its optical response is provided by the characteristic photoluminescence emission of nanostructured silica, as it will be discussed in more detail below.

### Light transmitted by a single *P. strigosum* valve: wavelength dependence

**Figure 5:**
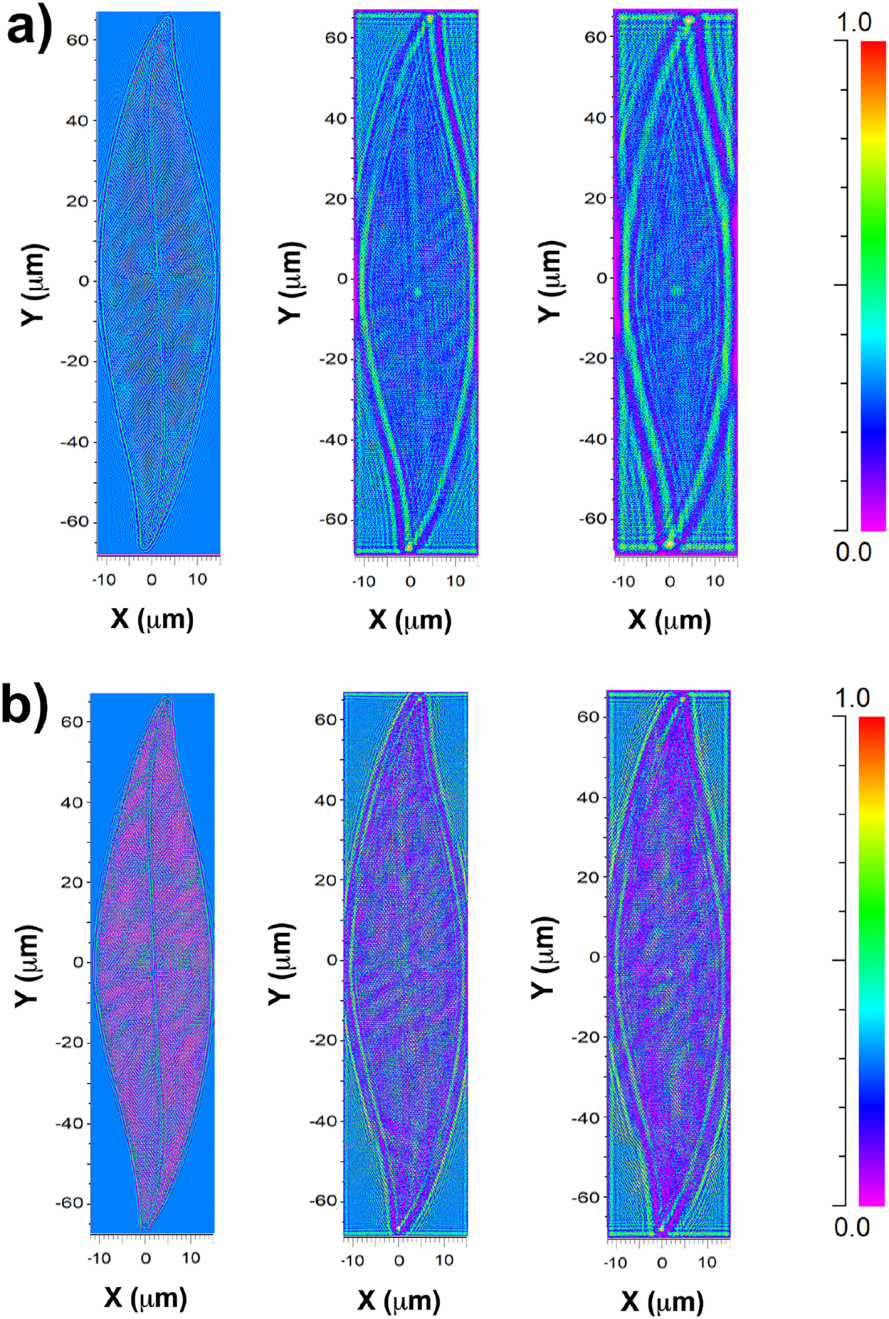
Color-encoded intensity transmitted by a single *P. strigosum* valve evaluated by WA-BPM for an incident wavelength of $\lambda =633$ nm (**a**) and $\lambda =280$ nm (**b**) at different distances along the optical axis $\hat{\textbf{z}}$: $z=0.4$ µm immediately after the valve (first column); $z=5$ µm (second column); $z=10$ µm (third column). Incident intensity: 0.3 (a.u.). Fields propagating in air.

The fate of light inside the cell can be inferred starting from the assessment of the intensity transmitted by the valve and its spatial distribution as a function of the incoming wavelength^42,50^. The relocation of radiation induced by diffraction, light deflection caused by refraction, and silica absorption strongly rely on the spectral content of the incoming optical field. In Fig. 5, numerically evaluated transmitted intensity maps using the wide-angle beam propagation method (WA-BPM, see Methods and Supplementary information) are depicted at various positions along the propagation axis $\hat{\textbf{z}}$ for two different incoming wavelengths. The valve is oriented normally to the direction of the incoming field propagation and spans from $z=0$ µm to $z=0.4$ µm. Considering an incident plane wave with a wavelength of $\lambda =633$ nm (Fig. 5a), the field immediately after the valve (first column) appears largely unchanged due to the high transparency of silica in the visible spectral range, as indicated by the color bar encoding the intensity values. The second and third columns represent the intensity evaluation at $z=5$ µm and $z=10$ µm, respectively. Transmitted intensity shows local enhancements corresponding to the pores of the silica matrix (diffraction contribution), the central nodule (refraction contribution), the valve edges (scattering and diffraction contributions), and apices (scattering and refraction contributions). These patterns of enhanced light intensity tend to spread as the field is assessed farther from the valve. Given that the entire frustule can reach approximately 20 µm in height, it is reasonable to conclude that a significant portion of visible light interacting with the valve is redirected towards the interior of the cell. Similar results are observed across the remaining part of the visible spectrum. Simulations for $\lambda =532$ nm and $\lambda =460$ nm are provided in the Supplementary Information (Fig. S2), along with the spatial distribution of transmitted intensity in the *XZ* plane (Fig. S3). As the wavelength decreases towards the ultraviolet B region (UV-B) ($\lambda =280-315$ nm), biosilica absorption becomes prominent, as shown in Fig. 5b for $\lambda =280$ nm. The intensity is substantially attenuated as the field propagates through the valve (first column), and regions of enhancement are less extensive for $z>0$ µm (second and third columns corresponding to $z=5$ µm and $z=10$ µm, respectively). The spot corresponding to refraction induced by the central nodule is scarcely detectable in this case. However, absorption by biosilica is not the sole mechanism reducing exposure to UV-B radiation, as, for sufficiently short wavelengths, diffraction-induced light divergence is less pronounced, and confinement effects can only occur very far from the valve or may not occur at all (see Fig. S3 in Supplementary Information).

**Figure 6:**
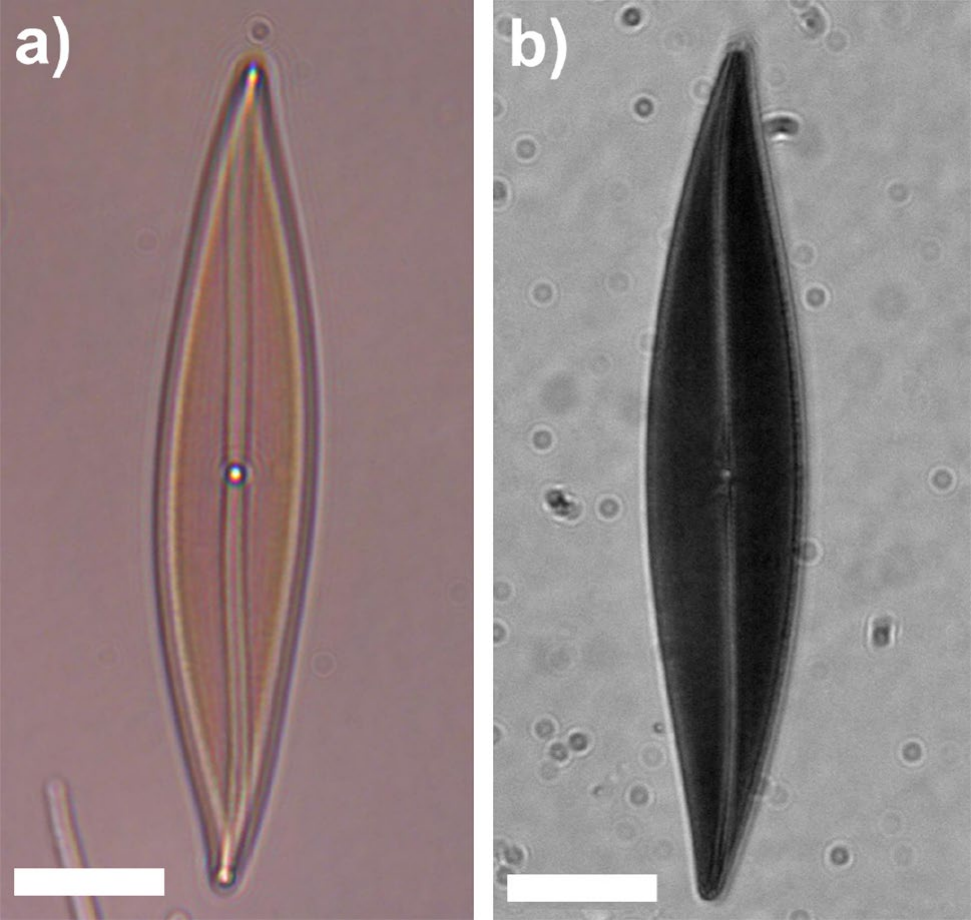
Transmission micrographs of a single *P. strigosum* valve when irradiated by PAR (**a**, $\lambda =$400–700 nm) and UV-B radiation (**b**, $\lambda =$280–315 nm). Scale bar: 25 µm.

The numerical simulations were based on a simplified CAD model obtained by extruding a two-dimensional image of a single valve (see Methods and Supplementary Information). This model does not consider the presence of the mantle (the downturned side surrounding the valve face). However, experimental evaluation of the spatial distribution of the intensity transmitted by a single valve aligns well with the computational results shown above when accounting for the distance between the valve face and the mantle edges, as illustrated in the micrographs in Fig. 6. These micrographs were acquired in transmission when the valve was irradiated with PAR (Fig. 6a) and UV-B radiation (Fig. 6b), respectively. In both cases, the incoming radiation is collimated and impinges normally onto the sample. When the mantle edges are in focus, the distance between the focal plane of the acquired image and the valve face equals the mantle height (up to about 3 µm), and a bright spot is detectable in the case of PAR illumination, consistent with the transmitted intensity maps obtained from numerical simulations. At this distance from the valve, the ratio of the bright spot intensity $I_{bs}$ to the incident intensity $I_0$ equals $1.89\pm 0.02$. Light scattered by valve apices and edges is also detectable. In the case of exposure to UV-B, most of the valve appears opaque (see Fig. 6b) due to absorption by the hydrogenated biosilica, and even the brighter regions exhibit attenuated intensity, with the $I_{bs}/I_0$ ratio in this case equal to $0.71\pm 0.02$.

**Figure 7:**
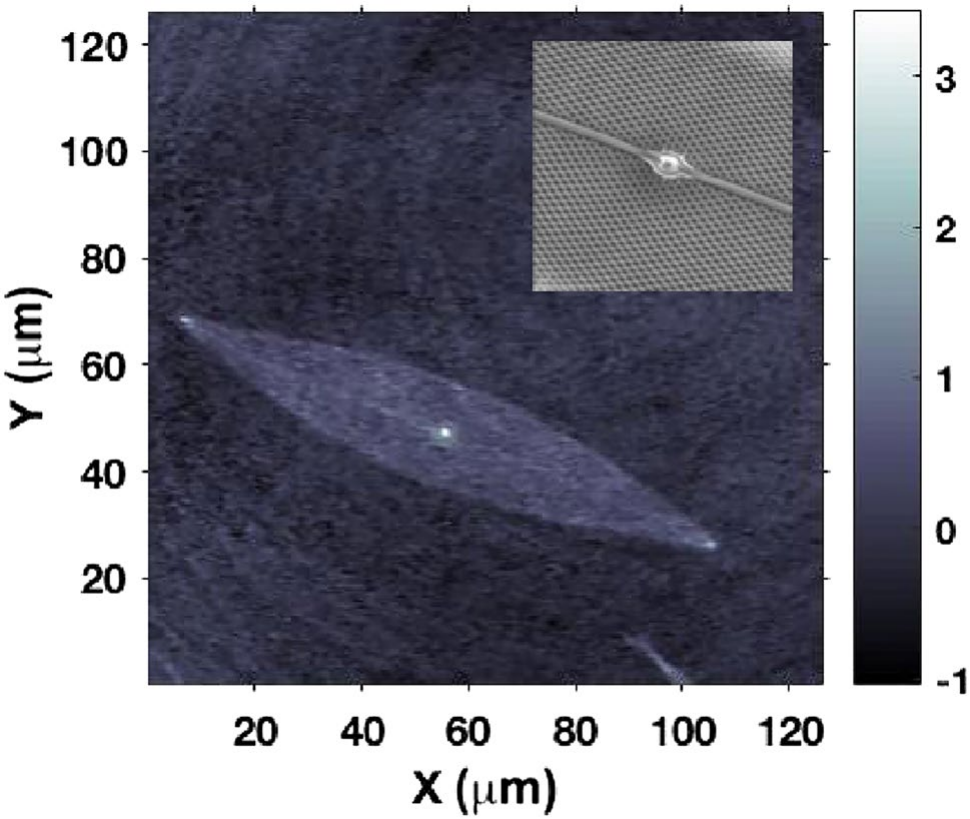
Phase map reconstructed at a distance $z=5$ µm from a valve irradiated by a laser beam at $\lambda =660$ nm. The bright spots at the center and the apices of the valve correspond to higher values of the optical path length respect to the surrounding area. Phase is expressed in radiants. Inset: a detail of the central area of the valve (inner layer) as detected by SEM.

Digital holography enables the reconstruction of both the amplitude and phase of the optical field interacting with a single valve. Figure 7 displays the phase map reconstructed at a distance of $z=5$ µm from the valve when illuminated by a laser beam with a wavelength of $\lambda =660$ nm. In this context, bright regions correspond to high values of the optical path length $n\cdot l$ compared to the surrounding area, where *n* is the refractive index of the medium, and *l* is the distance traveled by light. The central bright spot aligned with the axial position of the nodule is associated with the higher density of the nodule itself compared to the rest of the valve, which is porous (see the inset in Fig. 7). This induces light refraction and focusing in the intense hot spot predicted by numerical simulations and detected by transmission imaging. Similar conclusions apply to the valve apices, which also appear bright in the phase map.

As observed in various centric^35^ and pennate^42^ species, the morphology and composition of *P. strigosum* valves are designed to facilitate the transmission and collection of PAR, making it available for the photosynthetic processes inside the cell, while efficiently screening the diatom from potentially detrimental UV-B radiation. A further mechanism contributing to cell protection from harmful radiation will be described in the following section.

### *P. strigosum* luminescence: main contributions and emission channels

Diatom luminescence involves various cell compartments, and is primarily attributed to frustule photoluminescence^51^ and autofluorescence from chlorophylls^52^ and lipids^53^. Cathodoluminescence and electroluminescence can also be induced in diatom frustules, the latter in particular after metabolic insertion of semiconducting composites^54^. Frustule photoluminescence is affected by growth conditions, being especially enhanced in silicon starvation^52,55^, and it has been exploited as optical transducing mechanism in several sensing^33^ and biosensing^56^ schemes.

The origin of frustule photoluminescence lies in surface defects within the porous, hydrogenated amorphous silica matrix. These defects cause the presence of energy states within the silica bandgap which are accessible to electrons after absorption of photons in the ultraviolet-visible spectral range (UV-VIS)^55^. Electrons can subsequently decay with the emission of a photon with lower frequency. This emission mechanism is quite different from the one typical of porous silicon, which is primarily based on quantum confinement processes^57,58^. Among the surface defects which are considered as source of visible photoluminescence in porous silica, $\equiv$Si-H groups are likely responsible for green emission (around $\lambda =550$ nm), emission in the red spectral range (around $\lambda =700$ nm) is mainly attributed to oxygen dangling bonds (also known as non-bridging oxygen hole centers, NBOHCs) while surface silanol groups ($\equiv$Si–OH) are often associated to emission in the blue spectral range^57,59,60^.

**Figure 8:**
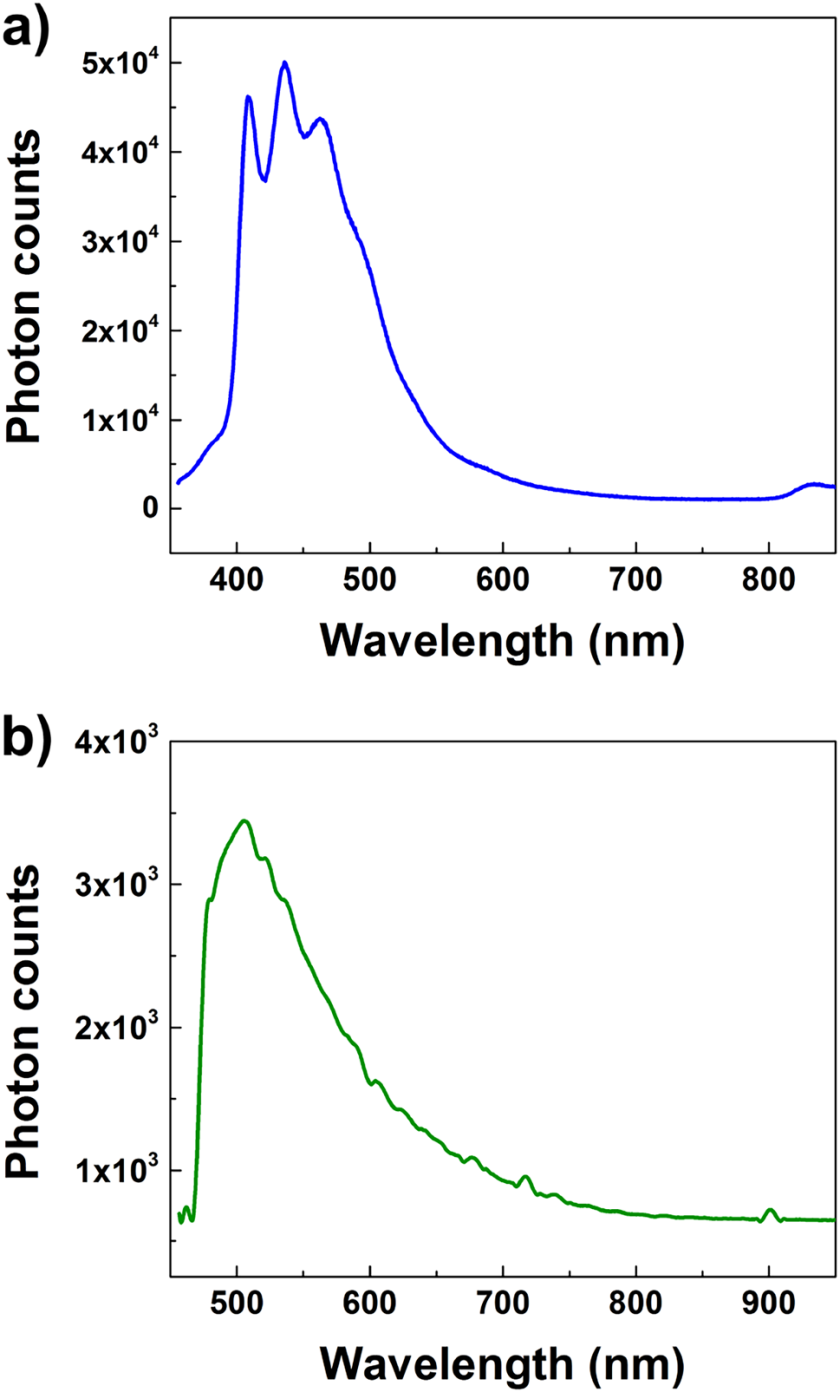
Photoluminescence emission spectra of a sample of dense frustules deposited onto a silicon wafer after excitation at $\lambda =325$ nm (**a**, $P_0=$ 3.2 mW, $t=0.1$ s) and at $\lambda =442$ nm (**b**, $P_0=$ 3.4 mW, $t=1$ s). The spectra have been acquired from the same sample. $P_0$: incident laser power; *t*: integration time.

In order to quantify the relative efficiency of different emission channels, we acquired the photoluminescence spectra of a dense sample of *P. strigosum* frustules deposited onto a silicon chip after excitation at two different wavelengths coming from a continuous wave He-Cd laser source. After irradiation at $\lambda _{ex}=325$ nm (UV-A, incident power $P_0=3.2$ mW, integration time $t=0.1$ s), most of the emission resulted in the violet-blue-cyan spectral range, with three convolved peaks at $\lambda _1=407$ nm (violet), $\lambda _2=436$ nm (violet), and $\lambda _3=464$ nm (blue), as can be seen in Fig.8a. After excitation at $\lambda _{ex}=442$ nm (incident power $P_0=3.4$ mW, integration time $t=1$ s), the emission results peaked around $\lambda \simeq 505$ nm (green, see Fig.8b). Taking into account the involved power and integration time, the latter emission channel results about two orders of magnitude less efficient respect to the one triggered by UV excitation, as can be deduced by evaluating the detected photon counts. Similar results have been previously obtained for *Coscinodiscus wailesii*^35^ and *Ctenophora pulchella*^42^ frustules.

It is interesting to understand if and at which extent the radiation emitted by the frustule is spectrally superimposed to the absorption features of diatom chromophores. Diatom main pigments include chlorophyll a (Chl-a), chlorophyll c (Chl-c) (both involved in light harvesting for photosynthesis), and carotenoids (fucoxanthin, $\beta$-carotene and the xanthophylls - diatoxanthin and diadinoxanthin), and in high radiation conditions a minor contribution of violaxanthin, antheraxanthin, and zeaxanthin can be detected^61^. Carotenoids are mostly implicated in photoprotection, with the exception of fucoxanthin, which transfers excitation energy very efficiently to Chl-a and plays a major role in light-harvesting complexes^45,62^. Chl-c is characterized by a strong blue absorption band and a weaker band in the red region (more pronounced in Chl-a, which also absorbs in the violet-blue spectral range).^62,63^. Carotenoids exhibit intense absorption between 400 and 500 nm, with exception of fucoxanthin whose broad absorption between 460 and 570 nm covers the gap left by Chl-a and Chl-c in the green spectral range^64^. Thus, the photoluminescence emission of *P. strigosum* frustules after UV-blue excitation entirely falls within the absorption spectra of the main diatom pigments. In clear oceanic waters, UV-violet-blue radiation is able to reach depths down to several tens of meters (in particular 60–70 m for UV-B^45,65^). It is likely that blue and green radiation emitted isotropically after UV-blue excitation is partially coupled to diatom frustule and, ultimately, partially transferred to plastids^23^. The emission channel triggered by exposure to UVR is of particular interest, in that radiation intrinsically detrimental for DNA (especially through formation of dimeric photoproducts between adjacent pyrimidines^35,42,66^) is efficiently converted in PAR. Being involved in the capability to downshift the high energy UV photons into low energy visible photons suitable for performing photosynthesis, photoluminescence represents a further mechanism by which diatom frustules screen the living cell from harmful radiation, in conjunction with silica absorption and spatial relocation of the optical field induced by diffraction. In this scenario, the observed strengthening of photoluminescence intensity in case of silicon starvation^52,55^ may represent a compensation mechanism by which wavelength conversion balances the lower content of UVR-absorbing silica.

### Interaction of UV-VIS radiation with the living cell in an aquatic environment

The interaction of *P. strigosum* diatoms with UV-VIS radiation is characterized by pronounced dynamism, both in their ability to move in reaction to changes in light exposure^67^ and in their diverse response to varying spectral content. In the latter case, as seen in previous sections, the frustule plays a fundamental role in spatial localization, coupling, spectral conversion, enhancement, or attenuation of optical radiation. Of particular interest from a biological point of view is the interplay between living diatoms and UVR. Most diatoms are characterized by a low content of mycosporine-like amino acids (MAAs)^68,69^, pigments usually present in phytoplankton and able to protect the cell by partially absorbing UV-A and UV-B radiation. More precisely, MAAs are distinguished by an absorption spectrum ranging between about 310 and 360 nm^70^, which, in any case, mostly falls outside the more detrimental spectral range of UV-B. Nevertheless, several diatom species can tolerate high doses of UVR with small effects on growth rates and low induction of DNA lesions^71–73^. This ability has been previously correlated with the screening mechanisms induced by diatom biosilica, with numerical simulations and experiments conducted on both bare frustules^35,38^ and living cells in their aquatic environment^42^.

In order to test the response of *P. strigosum* cells to significant UVR doses, we exposed different cultures to distinct irradiation treatments for 7 days (see Table 1 and the Methods for definitions of P, PA, and PAB treatments). For control, P and recovery treatments the light cycle was 16 h light : 8 h dark. For PA and PAB treatments, the total light cycle was still 16 h light: 8 h dark, but during the last 3 hours, the cells were exposed to PAR only. The results of the different irradiation regimes are reported in Table 2. Over 7 days, the growth rates were low and similar between the treatments, resulting consistent with each other (within one standard deviation). The growth rates could be considered the sum of all treatment effects on the cells over time. The maximum quantum yield ($F_V/F_m$, see Methods), was most affected in the PAB treatment, thus, the lower ratio was attributed to higher stress due to UV-B radiation. However, $F_V/F_m$ doubled after 24 h recovery in the same irradiation conditions as in the control sample.

**Table 1:** Irradiation treatments used in the in vivo experiments. Light/dark cycles are specified in the main text.

*P. strigosum* culture	PAR (µmol photons m^-2^ s^-1^)	UV-A (W/m^2^)	UV-B (W/m^2^)
Control	40	0	0
P	120	0	0
PA	120	5	0
PAB	120	5	1–1.4

**Figure 9:**
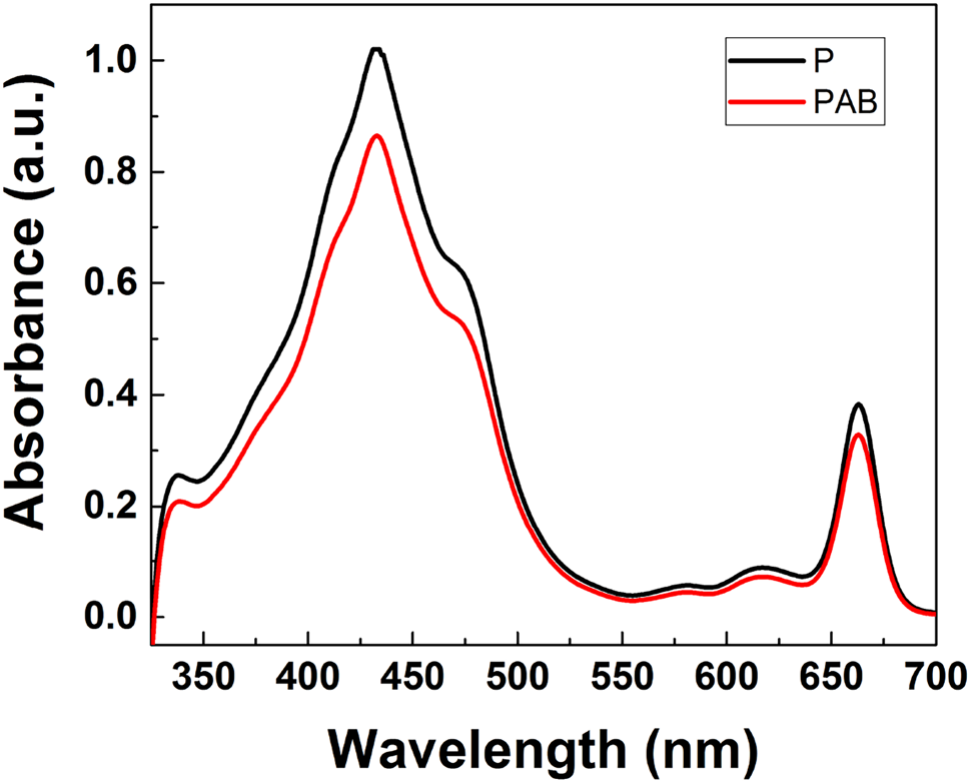
Absorbance spectra of a P-treated sample (black line) and a PAB-treated sample (red line) of *P. strigosum* revealing low contribution around $\lambda =334$ nm (ascribable to MAAs) if compared to absorption by other pigments.

This indicates a dynamic photoinhibition. Similar results were observed for benthic diatom communities dominated by *Pleurosigma obscurum*^73^ and *Gyrosigma fasciola*^74^. MAAs content in the cells was relatively poor, as can be deduced from the spectrophotometric chromatogram reported in Fig. 9, where the relative absorbance around $\lambda =334$ nm (mainly ascribable to porphyra-334 and shinorine, the main MAAs found in diatoms) has to be compared to that of other pigments (in this case mainly carotenoids and Chl-a and c). In any case, it is still worth noticing that even if MAAs content were higher, it would not guarantee protection from most of the UV-B spectral range. Furthermore, excessive energy can be modulated through the thermal dissipation by de-epoxidation of the xanthophyll cycle pigments diadinoxanthin to diatoxanthin^75^. Already in the P treatment, a higher ratio of diatoxanthin relative to the pool of diadinoxanthin and diatoxanthin was observed (about 0.48 for both P and PAB treatments, see Table 2). Also, the total sum of carotenoids (fucoxanthin, cis-fucoxanthin, diadinoxanthin, diatoxanthin, and betacarotene) relative to chlorophylls (Chl-a and Chl-c) was similar between the treatments, approximating unity (about 1.08 for P and 0.96 for PAB). The lack of treatment effects for the xanthophyll cycle conversion could be due to a too long handling time before freezing, as the conversion of diadinoxanthin to diatoxanthin could happen within seconds or a few minutes. In conclusion, *P. strigosum* seems relatively unaffected by the UV radiation applied.

**Table 2:** Results of different irradiation treatments on *P. strigosum* cells in terms of maximum quantum yield ($F_V/F_m$), specific growth rate per day ($\mu$), diatoxanthin (*DT*) relative to the pool of diadinoxanthin and diatoxanthin ($DD+DT$), and the total sum of carotenoids (*TC*) relative to the sum of Chl-a and Chl-c (Chls). The same quantities have been evaluated after 24 h recovery in the same irradiation conditions as in the control sample. Standard deviation is reported in brackets.

*P. strigosum* culture	$\textbf{F}_{\textbf{V}}/\textbf{F}_{\textbf{m}}$	$\varvec{\mu }$ (day^-1^)	$\frac{{\textbf{DT}}}{{\textbf{DD}}+{\textbf{DT}}}$	$\frac{{\textbf{TC}}}{{\textbf{Chls}}}$
Initial (all)	0.57 (0.05)	NA	NA	NA
7 days irradiation treatment				
Control	0.57 (0.06)	0.08 (0.02)	0.34 (0.14)	1.1 (0.2)
P	0.42 (0.07)	0.05 (0.04)	0.48 (0.04)	1.07962 (0.00003)
PA	0.49 (0.03)	0.07 (0.03)	NA	NA
PAB	0.22 (0.11)	0.089 (0.014)	0.476 (0.013)	0.96 (0.04)
24 h recovery				
Control	0.57 (0.05)	NA	NA	NA
P	0.58 (0.06)	NA	0.2462 (0.0007)	0.98 (0.07)
PA	0.60 (0.06)	NA	NA	NA
PAB	0.45 (0.18)	NA	0.30 (0.04)	0.97 (0.10)

**Figure 10:**
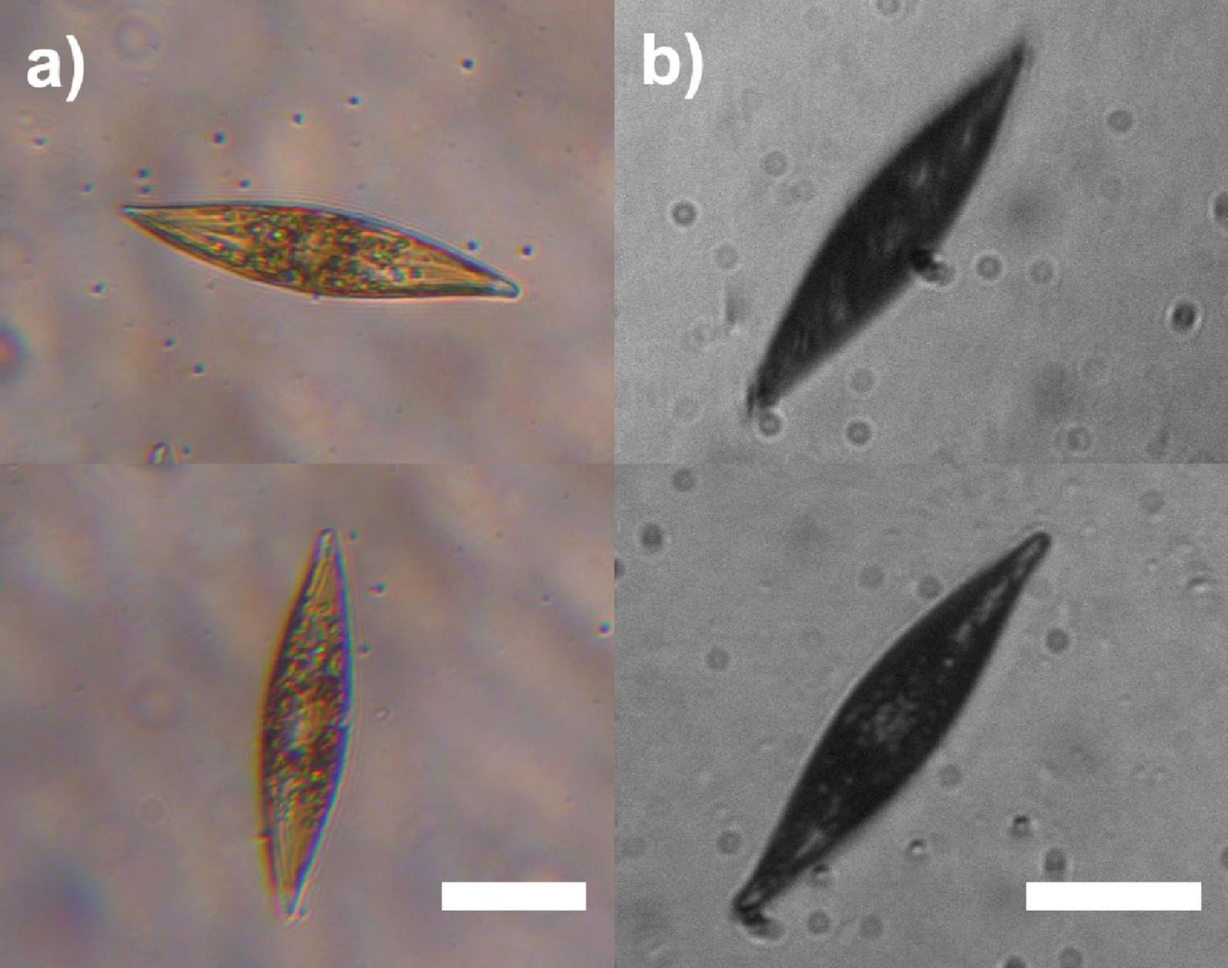
Transmission micrographs of single living *P. strigosum* cells immersed in their growth medium when illuminated by PAR (left column, $\lambda =$400–700 nm) and UV-B radiation (right column, $\lambda =280-315$ nm). Scale bar: 50 µm.

**Figure 11:**
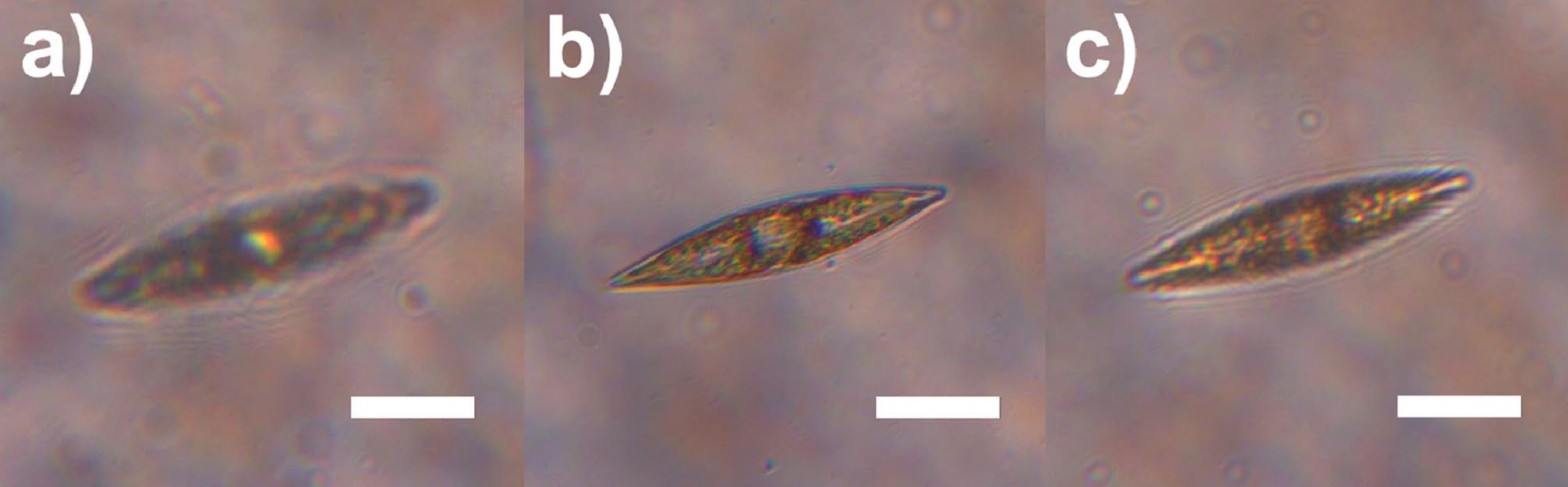
Transmission micrographs of a single living *P. strigosum* cell immersed in its growth medium when illuminated by PAR ($\lambda =$400–700 nm) at three different positions along the propagation axis $\hat{\textbf{z}}$: far-field in backward direction ($z\ll 0$ µm, **a**); focal plane ($z=0$ µm, **b**); far-field in forward direction ($z\gg 0$ µm, **c**). Scale bar: 50 µm.

**Figure 12:**
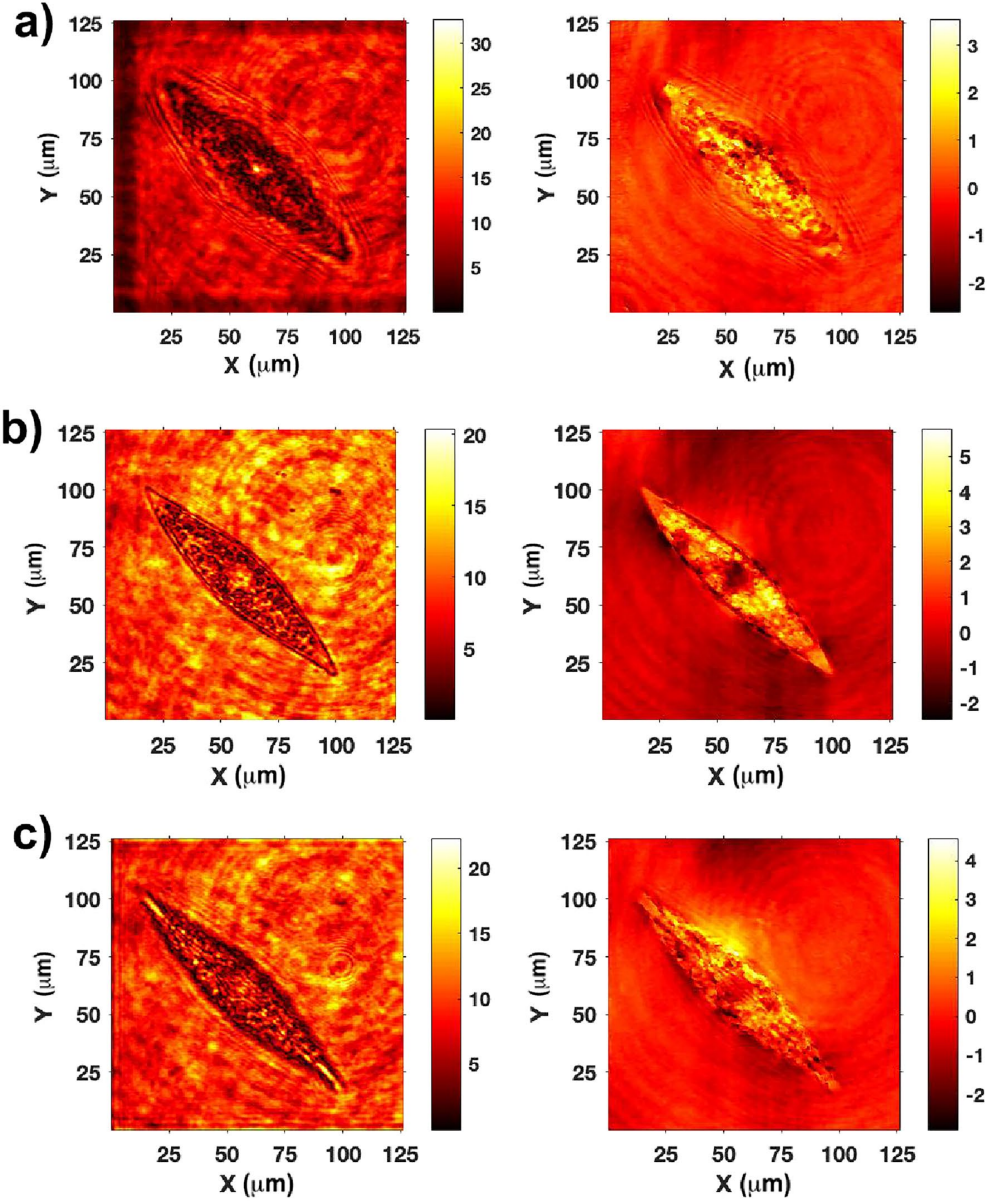
Intensity (left column) and phase (right column) maps relative to a single living *P. strigosum* cell immersed in its growth medium numerically reconstructed at different distances along the propagation axis $\hat{\textbf{z}}$ after acquisition of a hologram: $z=-30$ µm (**a**), $z=0$ µm (**b**), and $z=20$ µm (**c**). Irradiation wavelength: $\lambda =660$ nm. Intensity is expressed in arbitrary units while phase in radiants.

From a qualitative standpoint, when irradiated by PAR in their own growth environment (enriched seawater F/2 medium^76,77^), the living cells appear quite transparent, and their inner structure, mainly occupied by plastids, is clearly visible (see Fig. 10a). Conversely, when illuminated by UV-B radiation and observed in transmission by a UV-sensitive camera, the cells appear dark, primarily as a consequence of absorption by biosilica (see Fig. 10b). In any case, even in the regions where they appear less opaque (light gray areas within the cells in Fig. 10b), the transmitted intensity is about 30-40% less intense compared to the incident one (see Fig. S4 in Supplementary Information). Only in the case of irradiation by PAR, singular light patterns are detectable in the far-field (see Fig. 11). In particular, in the backward direction, a bright spot aligned with the position of the nodule along the optical axis can be observed (Fig. 11a), most likely attributable to radiation backscattered by the nodule itself. In the opposite direction (Fig. 11c), light confined and scattered along the sternum is more evident towards the edges. This contribution may be related to light guided by the sternum and able to seep inside the cell through the associated evanescent fields^23^. Similar light patterns have been detected by digital holography measurements performed at $\lambda =660$ nm (see Fig. 12). Looking at the intensity maps reconstructed in the far field for negative ($z=-30$ µm, Fig. 12a, left column) and positive ($z=20$ µm, Fig. 12c, left column) values of *z*, the same spatial light distributions observed by transmission imaging are indeed retrieved. In the phase map relative to the focal plane ($z=0$ µm, Fig. 12b, right column), small values of the optical path length in correspondence to the central area of the cell (dark region in the map) with respect to the surroundings are obtained. This is compatible with the lower values of the nucleus and nucleolus refractive indexes ($n=1.355-1.365$ and $n=1.375-1.385$ respectively^78,79^) when compared to the one of plastids ($n\simeq 1.51$^80,81^), which occupy most of the cell volume.

Observations of living individuals immersed in their aquatic environment are thus consistent with the light manipulation mechanisms of the frustule described above. In particular, the transmission of radiation inside the cell and its coupling to plastids, mediated by frustule components, is favored for visible radiation, while UVR is mostly extinguished by biosilica.

## Conclusions

The extraordinary diversity of diatoms reveals itself in the huge variety of shapes and geometries of the frustules observed through different genera and species. In the present work, we identified single components of *P. strigosum* frustule able to interact with optical radiation and act as diffractive, refractive, scattering, waveguiding, and photoluminescent elements. We can assign to the porous matrix of the valve, characterized by a regular modulation of the refractive index, the role of a diffraction grating, facilitating the coupling with incident light and the spatial relocation of radiation inside the cell according to wavelength; the central nodule plays the role of a microlens; photoluminescence of nanostructured silica promotes frequency down-conversion of high-energy optical fields. The overall effect of these components is an effective collection of PAR and a simultaneous screening of the cell from noxious UVR. UVR shielding, in particular, results from a collective action, including biosilica absorption, intensity relocation via diffraction, and wavelength conversion through photoluminescence. The impact on living organisms of these combined effects, was assessed by measuring growth rates and other vital parameters following 7-days exposure to various irradiation regimes, including UVR. The observed parameters resulted relatively unaffected by UV irradiation or characterized by dynamic, reversible modifications. The optical influence of the valves on individuals in their aquatic environment was also analyzed, demonstrating effective PAR trapping beneficial for the photosynthetic process.

Besides photosynthesis and photoprotection, a potential link between light modulation exerted by the *P. strigosum* frustule and phototaxis of the living organism is worth investigating in the future. Unlike focusing, diffraction, and photoluminescence, light propagation along the sternum is hardly relatable to light harvesting. The evanescent electromagnetic fields associated with the modes guided by the sternum can penetrate only a few hundred nanometers inside the protoplasm, indeed, and it would be interesting to investigate possible signal cascades involved in phototaxis and triggered by the radiation captured by the frustule.

A diatom culture can be envisioned as a biological nano-factory capable of producing, at high rates and with a noticeable level of reproducibility, self-replicating complex dielectric nanostructures that are challenging to achieve even with the most recent nanolithographic techniques. In addition to the direct utilization of diatom biosilica in nanotechnology, diatom frustules could serve as a wellspring of inspiration in the creation of innovative technologies. Exploring the fundamental aspects of diatoms’ optical properties might open avenues for the development and manufacturing of innovative, bio-inspired optoelectronic devices, enabling the simultaneous manipulation of light through various operational channels.

## Methods

### Sampling, culture conditions, frustule cleaning

Samples containing *P. strigosum* cells were collected from a shallow softbottom at the Swedish west coast (Tjärnö island, Sweden; 58^∘^ 52′ N; 11^∘^ 09′ E). After isolation, cells were kept in 50 ml Nunc flasks filled with F/2 medium in a climate chamber at 16 ^∘^C and 40 µmol photons m^-2^ s^-1^ (16 h light and 8 h darkness). The light was provided by white fluorescent tubes (Philips Master, TL-D 58W/840, Reflex Eco, Holland) and was adjusted using a cosine quantum sensor (LI-COR, LI-1400, USA). For frustule cleaning, the samples were boiled in 35% hydrogen peroxide for 15 min to remove all organic content in the frustule. Clean frustules were then rinsed several times in deionized water to remove salt residuals.

### Microscopy

Bright field, dark field, and crossed-polarization images of single cleaned valves deposited onto a fused quartz slide have been acquired by a Leica DM6000 microscope (Leica Microsystems, Wetzlar, Germany) with a 50X objective in dry medium.

Fluorescence images of single cleaned valves deposited onto a silicon wafer were acquired by using a Leica DM6M microscope (Leica Microsystems, Wetzlar, Germany) equipped by a solid-state fluorescence light source (Leica SFL4000) and controlled via LAS X software (Leica Application Suite; rel. 3.0.13). The imaging was performed with a 50X objective in dry medium, exciting the fluorescence emission by two LED modules emitting at $\lambda =365$ nm and $\lambda =470$ nm, respectively.

For scanning electron microscopy (SEM) observations, a cleaned diatom aliquot was filtered on 1 mm Nucleopore Whatman filter and left to dry on an aluminum stub covered with conductive and adhesive black carbon disks before coating with gold palladium alloy. A LEO Ultra 55 SEM (Chalmers University, Gothenburg, Sweden) was used for examining and imaging. Examination was made on 8 kV at a distance of 8 mm.

To characterize plastids, cells were examined using confocal laser scanning microscopy (CLSM) at Chalmers University, Gothenburg, Sweden (Nikon Ti-E with A1-DUG DU-Hybrid Detector Unit with PMT and A1 Filter Cube 595/50 700/75; Nikon LU-N4 laser box: Spectral Physics 640 nm; CFI Apo $\lambda$S 60X Oil objective with nano-crystal coat). The recorded 3D-image stacks were visualized as 2D maximum intensity projections.

### Numerical simulations

The propagation of the optical field through a single *P. strigosum* valve at different wavelengths has been numerically evaluated by making use of the Wide-Angle Beam Propagation Method (WA-BPM) as implemented in the RSoft CAD Beamprop environment (Synopsis). The simulations were run on a DELL workstation (Xeon bi-processor with $2\times 14$ cores, 512 GB RAM, graphic board NVIDIA QUADRO K6000). Refractive index maps have been retrieved starting from top view SEM images of the inner and outer layers of a single valve, thus reproducing the deviation from perfect periodicity which is characteristic of a real frustule. The refractive index maps of the two layers were then extruded and superimposed in order to obtain a realistic 3D CAD model of the valve. The incoming field is given by a plane wave at a given wavelength and is normally incident onto the valve. The spatial distribution of the transmitted intensity has been evaluated taking into account silica dispersion and absorption. More details on WA-BPM and on the CAD model used in the numerical simulations can be found in Supplementary Information.

### Transmission imaging

Transmission imaging in different spectral ranges on single valves and on living cells immersed in their growth medium have been performed by making use of a UV-VIS source (Hamamatsu Photonics, Hamamatsu, Japan, model L10290), including a deuterium lamp ($\lambda =$200–400 nm) and a tungsten halogen lamp ($\lambda =$400–1100 nm) which can be used independently. A band-pass filter (Asahi Spectra, Torrance, CA, USA, UV-B filter) was used for measurements in UV-B spectral window ($\lambda =$280–315 nm). Radiation is emitted through an optical fiber transparent in $\lambda =$200–700 nm spectral range (Hamamatsu, A7969 anti-solarization fiber) provided with a quartz collimator (Lot Oriel, LLZ010). For measurements on cleaned frustules, sparse valves have been deposited onto a fused quartz microscope slide (UQG optics, FQM-7521, transmission $>90$% between $\lambda =280$ nm and $\lambda =2$ µm) and invested by the collimated beam. The slide was mounted on a XYZ translational stage (Thorlabs, PT3/M) in order to select the single valve. Transmitted radiation was collected by a microscope objective (Zeiss, Oberkochen, Germany, 50X Epiplan, NA 0.7 for measurements in visible spectral range; Thorlabs, LMU-40X-UVB, NA 0.49, AR coatings in $\lambda =240-360$ nm range for measurements in UV-B) mounted on a micrometric translation stage (Thorlabs, PT3A/M) in order to acquire images at different distances from the valve. The collected, expanded radiation was finally sent to a CMOS camera (IDS, UI-1240SE-C-HQ, quantum efficiency: $>40$% in red, green, and blue spectral windows) in case of visible spectral range and to a UV-sensitive CCD camera (Hamamatsu, C8484-16C, quantum efficiency: 25–33% for $\lambda =$280–315 nm) for measurements in UV-B. In the latter case, a further UV-B bandpass filter has been inserted in the inlet of the CCD camera in order to avoid the detection of undesired visible photoluminescence induced by UV excitation.

For measurements on living cells, the irradiated sample is given by a suspension of microalgae immersed in their growth medium (F/2) and injected into a chamber ($25 \times 25$ mm wide, $\sim 100$ µm thick) obtained by sealing two UV fused silica coverslips (UQG optics, CFS-2525, transmission >90% between $\lambda =175$ nm and $\lambda =1.2$ µm). As for the bare valves, the use of micrometric translation stages allowed selecting single cells and collecting the transmitted radiation at different distances from the sample. In this case, the microscope objectives were characterized by lower magnifications in order to enlarge the field of view and follow the motile cells (Zeiss, Oberkochen, Germany, 20X Epiplan, NA 0.4 for measurements in visible spectral range; Thorlabs, LMU-20X-UV-B, NA 0.39, AR coatings in $\lambda =240-360$ nm range for measurements in UV-B).

### Digital holography

A laser beam emitted by a continuous wave, linearly polarized source (Torus solid state laser, Novanta Photonics, $\lambda =660$ nm, $P_{max}=700$ mW, coherence length $\simeq 100$ m) was collected by an objective lens (10X, NA 0.22) and splitted through an optical fiber coupler (F-PMC-633-50 single mode polarization maintaining fiber coupler, Newport, $1\times 2$, 70/30, FC/APC), giving rise to an object and a reference beam. After passing through the sample (a single valve onto a glass microscope slide in case of bare frustules; a single cell in its growth medium sandwiched between a microscope slide and a coverslip in case of living diatoms), the object beam was collected by a microscope objective lens (63X, NA 0.75) and then recombined with the reference beam by a beamsplitter. The resulting interference pattern was collected onto a CCD camera ($1024\times 768$ pixel array; pixel size $\Delta x=\Delta y=4.65$ µm). A half-wave plate has been used to rotate the polarization state of the object beam, in order to increase the fringe contrast of the interferogram. Aberrations introduced by the optics have been compensated by making use of a double exposure technique, consisting in the acquisition of a hologram on the sample under investigation and one on a reference surface in proximity of it. Aberrations can be compensated by numerically manipulating the two holograms^50^.

After the acquisition of the interference pattern, the amplitude and phase maps of the optical field which interacted with the sample can be numerically retrieved by applying a reconstruction algorithm described in detail in Supplementary Information.

### Photoluminescence spectroscopy

Steady-state photoluminescence (PL) spectra were acquired after excitation by a continuous wave He-Cd laser (KIMMON Laser System) emitting at $\lambda =325$ nm and $\lambda =442$ nm. PL radiation was collected at normal incidence to the surface of samples, constituted by dense aggregates of cleaned diatom valves deposited onto a silicon wafer, through an optical fiber, then dispersed by a spectrometer (Princeton Instruments, SpectraPro 300i) and detected using a Peltier cooled CCD camera (PIXIS 100 F). Long-pass filters with a nominal cut-on wavelength of 350 and 458 nm were used to remove the laser line at monochromator inlet for excitation at 325 and 442 nm, respectively.

### UVR treatments

Cells of *P. strigosum* were taken from the middle of logarithmic phase and transferred in 25 ml quartz flasks to the starting cell number of $1.7 \times 10^7$ to $1.8 \times 10^7$ cells L^-1^, with the addition of 10 ml fresh medium (number of replicas $n=3$). Background PAR was provided by the white fluorescent tubes used for culturing and described above, while UV radiation was provided by UV lamps (Q-Panel UV-A-340, 40 W). Narrower wavebands were selected by making use of quartz bottles covered with one of the following filters: Ultraphan transparent (280–700 nm; Digefra GmbH, Munich, Germany), Folanorm (320–700 nm; FolexGmbH, Dreieich, Germany), and UltraphanURUV Farblos (400–700 nm; Digefra GmbH, Munich, Germany) corresponding to the PAR+UV-A+UV-B (PAB), PAR + UV-A (PA), and PAR (P) treatments, respectively. UVR intensity was detected by a Solar Light PMA2100 radiometer equipped with the UV-A sensor PMA 2110 and the UV-B Sensor PMA 2106 (Solar Light, Philadelphia, PA). Adjusted UVR below the cutoff filters was 5 W m^-2^ for UV-A and 1–1.4 W m^-2^ for UV-B (13 h irradiation), while the experimental PAR was adjusted to 120 µmol photons m^-2^ s^-1^ (16 h irradiation) at 16 ^∘^C. The P, PA and PAB treatments lasted 7 days, and samples for the estimation of photosynthetic activity and growth rates were taken at the start and at the end of cultivation. Another set of flasks with *P. strigosum* cells was placed in a climate chamber under standard growth conditions (40 µmol photons m^-2^ s^-1^, 16 h light, 16 ^∘^C) and it was designated as a control ($n=3$).

The cell number was estimated using a gridded Sedgewick Rafter counting chamber under a light microscope (Zeiss, Axiovert 40, Germany), and specific growth rate per day ($\mu$) was calculated by the formula:

1 $$\begin{aligned} \mu =\frac{\ln \left( \frac{N_1}{N_0}\right) }{t_1-t_0} \end{aligned}$$

where $N_1$ and $N_0$ are the cell concentrations at the end and beginning of a period of time $t=t_1-t_0$ days.

### Chlorophyll fluorescence measurements

Photosynthetic efficiency was estimated starting from fluorescence of PSII, determined by using a pulse amplitude modulation fluorometer (Water PAM, Walz GmbH, Effeltrich, Germany). Prior to measurement, the number of cells was equalized. Immediately after sampling, the algal suspension was acclimated in darkness for 5 min at 16 ^∘^C and put in 5 ml Quartz cuvettes (Hellma, Müllheim, Germany). The suspensions were gently stirred using a small magnetic bean during the fluorescence measurements. The maximum quantum yield ($F_v/F_m$, ratio of variable to maximum chlorophyll fluorescence from photosystem II) was measured at time zero ($n=6$). After dark incubation, a pulse of weak, far-red light was applied to empty the electron pool from plastoquinone A ($Q_A$). The initial fluorescence ($F_0$) was measured with red measuring light ($\sim 0.3$ µmol photons m^-2^ s^-1^, $\lambda =650$ nm) and the maximum fluorescence ($F_m$) was determined using a 600 ms completely saturating white light pulse ($\sim 3500$ µmol photons m^-2^ s^-1^).

### UV-absorbing compounds and photosynthetic pigments

Cells were analyzed by spectrophotometry in order to detect the possible presence of the mycosporine-like amino acids (MAAs) usually found in (centric) diatoms, i.e. porphyra-334 and shinorine, both characterized by an absorption spectrum peaked at $\lambda =334$ nm. The cells were gently filtered onto a GF/F filter and the filter was put in a mixture of acetone:methanol 80:20. The extract was put in -20 ^∘^C for 24 h followed by 1 min ultrasonication using a 3 mm probe (Vibra-cell). The filtrate was purified using a 0.7 mm syringe filter and the solution was analysed spectrophotometrically between $\lambda =260$ nm and $\lambda =700$ nm.

Cell suspension of 5 ml was filtered on 25 mm GF/F filters and the filters were immediately frozen and stored at -80^∘^C until extraction. Prior to analyses, the filters were extracted in 1.5 ml acetone:methanol (80:20) and sonicated using a Vibra-cell sonicating probe, operating in pulses at 80% for 1 min. High performance liquid chromatographic (HPLC) analyses of the extracts were performed^82^, using an absorbance diode array-based detector (Spectraphysics UV6000LP). A 150 $\times$ 3.0 mm Phenomenex Kinetex 2.6-µ C18 100A column was used for separation. Pigments were identified by their retention time and absorbance spectra ($\lambda =400-700$ nm) and quantified using calibration standards, provided by DHI Water and Environment, Denmark.

### Supplementary Information

Supplementary Information.

## Data Availability

The data that support the findings of this study are available from the corresponding author upon reasonable request.
