# Supplementary material for: Multiple-pathways light modulation in Pleurosigma strigosum bi-raphid diatom
Source: Sci Rep. 2024 Mar 18;14:6476. doi: 10.1038/s41598-024-56206-y (PMC10948915; doi:10.1038/s41598-024-56206-y)
Supplement: Supplementary file 1 — Supplementary Information. [file 41598_2024_56206_MOESM1_ESM.pdf]

Multiple-pathways light modulation in *Pleuorosigma strigosum* bi-raphid diatom.
Supplementary Information.

Edoardo De Tommasi[1], Ilaria Rea[1], Maria Antonietta Ferrara[1], Luca De Stefano[1], Mario De Stefano[2], Adil. Y. Al-Handal[3], Marija Stamenković[3,4], and Angela Wulff[3]

[1]National Research Council, Institute of Applied Sciences and Intelligent Systems "E. Caianiello", Unit of Naples, Via P. Castellino 111, I-80131, Naples, Italy
[2]University of Campania "Luigi Vanvitelli", Department of Environmental, Biological, and Pharmaceutical Sciences and Technologies, Via Vivaldi 43, I-81100, Caserta, Italy
[3]University of Gothenburg, Department of Biological and Environmental Sciences, Box 463, 405 30 Göteborg, Sweden
[4]University of Belgrade, Department of Ecology, Institute for Biological Research "Sinisa Stankovic", Bulevar despota Stefana 142, 11060 Belgrade, Serbia

1 Wide-angle beam propagation method (WA-BPM)

Beam propagation method (BPM) is a computational tool routinely employed to simulate light propagation in waveguides and optical fibers, i.e. in paraxiality conditions (small angles with respect to the optical axis) and when a uniform refractive index along the direction of propagation of the field is considered. Since, in the case of diatom valves, the nano-porous structure induces diffraction and, after transmission through silica, the radiation propagates in a medium characterized by a different refractive index, it is mandatory to make use of a non-paraxial approximation of BPM.

Starting from the Helmoltz equation for a scalar field (i.e. neglecting polarization effects):

$$\nabla^2\phi + k^2(\mathbf{r}) = 0 \tag{S1}$$

with $E(\mathbf{r},t) = \phi(x,y,z)e^{-i\omega t}$ scalar electric field, $k = nk_0$ wavenumber (with $k_0 = \frac{2\pi}{\lambda}$ wavenumber in free space), and $n = n(x,y,z)$ refractive index spatial distribution, we can write the solution as:

$$\phi(x,y,z) = U(x,y,z)e^{-ik_r z} \tag{S2}$$

i.e. the electric field can be expressed as the product of a slowly varying envelope factor $U(x,y,z)$ and a rapid varying phase factor $e^{-ik_r z}$, where the reference wavenumber k_r takes into account the average phase variation of the field. We are assuming that the considered wave propagates primarily along z (i.e. we are considering, at first, paraxial conditions). We will also suppose, for now, that the amplitude varies slowly along z axis too. Inserting $U(x,y,z)e^{-ik_r z}$ into Eq. S1 we obtain:

$$\frac{\partial^2 U}{\partial z^2} + 2ik_r\frac{\partial U}{\partial z} + \frac{\partial^2 U}{\partial x^2} + \frac{\partial^2 U}{\partial y^2} + (k^2 - k_r^2)U = 0 \tag{S3}$$

Making use of the *slowly varying envelope approximation*:

$$\left|\frac{\partial^2 U}{\partial z^2}\right| \ll \left|2k_r\frac{\partial U}{\partial z}\right| \tag{S4}$$

we obtain the basic BPM equation:

$$\frac{\partial U}{\partial z} = \frac{i}{2k_r}\Big[\frac{\partial^2 U}{\partial x^2} + \frac{\partial^2 U}{\partial y^2} + (k^2 - k_r^2)U\Big] \tag{S5}$$

Specifying $U(x,y,z)$ at a plane $z = z_0$, we can iterate U along the z-axis using finite differences for the x and y derivatives.

A BPM variant which can take into account non-paraxial conditions (Wide-Angle Beam Propagation Method, WA-BPM), is known as the multistep Padé-based technique [1, 2, 3]. We can denote $\frac{\partial}{\partial z}$ with D, and, consequently, $\frac{\partial^2}{\partial z^2}$ with D^2. Eq. S3 can be now viewed as a quadratic equation to be solved for the differential operator D. This yields to the following solution for a first order equation in z:

$$\frac{\partial U}{\partial z} = ik_r(\sqrt{1+P} - 1)U \tag{S6}$$

with:

$$P \equiv \frac{1}{k_r^2}\Big(\frac{\partial^2}{\partial x^2} + \frac{\partial^2}{\partial y^2} + (k^2 - k_r^2)\Big) \tag{S7}$$

Even though it is restricted to forward propagation of the field ($z > 0$), the above equation is exact in that no paraxiality approximation has been introduced. The radical in Eq. S6 can be evaluated by using a Taylor expansion. The first order of the expansion leads to the standard, paraxial BPM, while higher orders lead to more accurate representations of the propagating field. However, expansion

**Padé order (m,n)**	$\mathbf{N_m}$	$\mathbf{D_n}$
(1,0)	$\frac{P}{2}$	1
(1,1)	$\frac{P}{2}$	$1+\frac{P}{4}$
(2,2)	$\frac{P}{2}+\frac{P^2}{4}$	$1+\frac{3P}{4}+\frac{P^2}{16}$
(3,3)	$\frac{P}{2}+\frac{P^2}{2}+\frac{3P^3}{32}$	$1+\frac{5P}{4}+\frac{3P^2}{8}+\frac{P^3}{64}$

Table S1: Low-order Padé approximants expressed in terms of the operator P defined in Eq.S7.

via Padé approximants is more accurate than Taylor expansion for the same order of terms. This approach leads to the following equation:

$$\frac{\partial U}{\partial z} = ik_r \frac{N_m(P)}{D_n(P)} U \tag{S8}$$

where N_m and D_n are polynomials in the operator P, and (m, n) is the order of approximation. Some of their low-order values are reported in Table S1, (1,0) order corresponding to paraxial BPM. Increasing the Padé order allows analyzing larger angles, higher refractive index contrasts, and more complex mode interference both for guided waves and fields propagating in free space. In this work, in order to balance accuracy and computation time, we made use of the $(2, 2)$ Padé order, corresponding to $N_2 = \frac{P}{2} + \frac{P^2}{4}$ and $D_2 = 1 + \frac{3P}{4} + \frac{P^2}{16}$.

2 CAD model of a single *P. strigosum* valve

CAD models used to simulate the propagation of optical fields through a single *P. strigosum* valve have been retrieved starting from SEM images of the inner and outer layers of the valve itself. The micrographs have been transformed into binary, bitmap images (see Fig.S1) from which refractive index maps have been derived, assigning the proper values of refractive index to the region occupied by silica and to the environment in which the valve is immersed (air). The refractive index maps have been then extruded and superimposed in order to obtain a 3D CAD model of the valve (overall thickness: 400 nm). The presence of the slits on the external side of the valve and of the bridges occluding the pores of the inner side are not resolved due to the magnification of the starting SEM images. Nevertheless, this does not represent an issue in terms of numerical evaluation of the fields since the linear dimension of these fine features (about 30 nm in width) is one order of magnitude smaller than the optical wavelengths, thus they do not induce detectable diffraction when hit by light.

The direction of propagation of the incoming plane wave was orthogonal to

the valve, and the transmitted intensity has been evaluated at different wavelengths, taking into account silica dispersion and absorption.

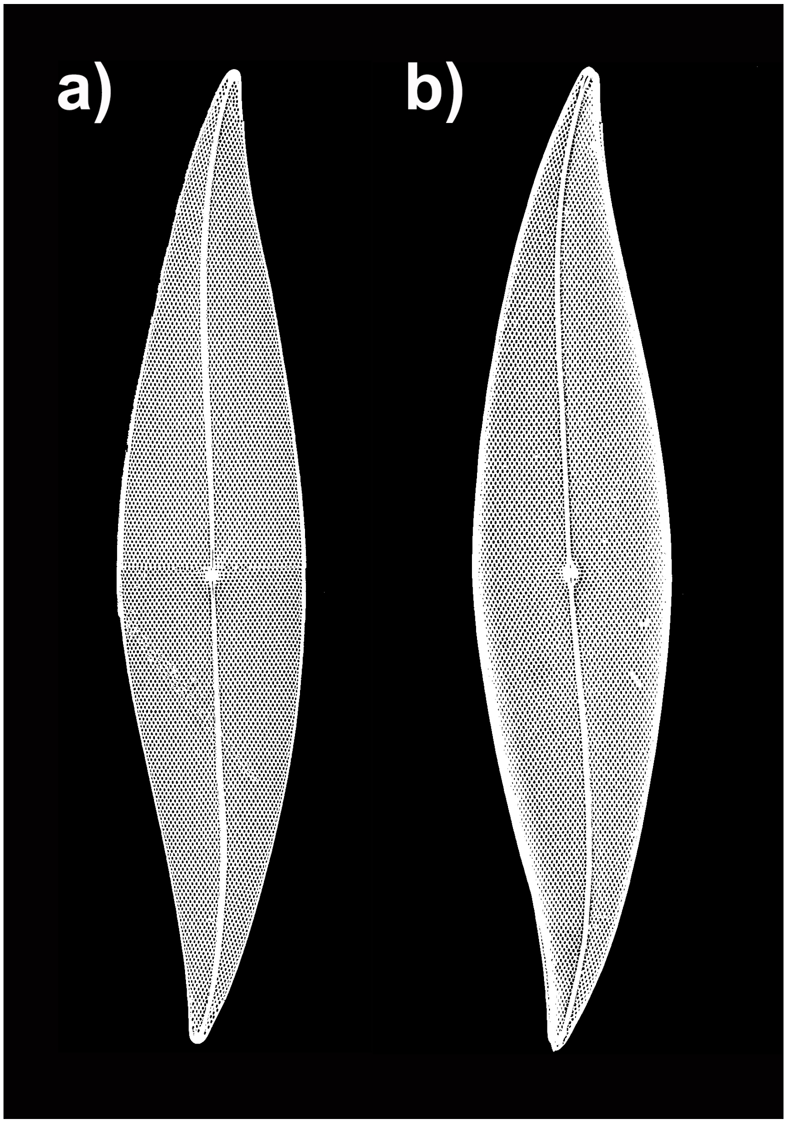

Figure S1: Binary images of the outer (**a**) and inner (**b**) layer of a *P. strigosum* valve as retrieved from SEM micrographs.

3 Amplitude and phase reconstruction by digital holography

The amplitude and phase of the optical wavefront diffused by the object under test can be mathematically retrieved by applying an image reconstruction procedure described in the following. Starting from the frequency spectrum of the acquired interference pattern in off-axis configuration, the first diffraction order is separated from the whole spatial frequency spectrum by a bandwidth filter and shifted to the origin of the plane. As a result, the spectrum of the object field (defined as $O(x,y) = |O(x,y)|e^{i\phi(x,y)}$, with $|O(x,y)|$ and $\phi(x,y)$ amplitude and phase, respectively, and x and y cartesian coordinates defining the plane of acquisition of the hologram) is obtained except for a constant [4]. The optical wavefront at different distances from the plane of acquisition can be reconstructed by applying the Fourier formulation of the Fresnel-Kirchhoff diffraction formula [5]. The Fresnel-Kirchoff integral, the lens transfer factor, and other operations can be otherwise replaced by operator algebra [6], which allows bypassing the cumbersome integral calculus. In this framework, the propagated field $O_{\text{prop}}(\xi,\eta)$ as a function of the initial field $O(x,y)$ can be expressed as [7]:

$$O_{\text{prop}}(\xi,\eta) = \exp(ikd) \times \left\{ \mathcal{F}^{-1}\left[\exp\left(-\frac{ikd\lambda^2}{2}(\nu^2+\mu^2)\right)\right] \cdot \mathcal{F}\left(O(x,y)\right)\right\} \quad \text{(S9)}$$

here $\mathcal{F}[f(x)]$ is the Fourier transform of the function $f(x)$, $k = \frac{2\pi n}{\lambda}$ (with n refractive index of the medium), ν and μ are spatial frequencies defined as $\nu = \frac{\xi}{\lambda d}$ and $\mu = \frac{\eta}{\lambda d}$, and d is the reconstruction distance. For digital reconstruction, Eq.S9 is implemented in a discrete form:

$$O_{\text{prop}}(m,n) = \exp(ikd)\left\{ \mathcal{F}_D^{-1}\left[-\frac{ikd\lambda^2}{2N^2\Delta^2}(U^2+V^2)\right] \cdot \mathcal{F}_D(O(h,j))\right\} \quad \text{(S10)}$$

where N and Δ are the number of pixels in both directions and pixel dimension, respectively, and m, n, U, V, h and j are integer numbers varying from 0 to $N-1$. The discretized Fourier transform is defined as:

$$\mathcal{F}_D\{g(j,l)\} = \frac{1}{N}\sum_{j,l=0}^{N-1} \exp\left[-\frac{2\pi i}{N}(mj+nl)\right] g(j,l) \quad \text{(S11)}$$

Intensity and phase distributions of the propagated field can be evaluated by using the following relations:

$$I_{\text{prop}}(m,n) = |O_{\text{prop}}(m,n)|^2 \quad \text{(S12)}$$

$$\phi_{\text{prop}}(m,n) = \arctan\frac{\text{Im}[O_{\text{prop}}(m,n)]}{\text{Re}[O_{\text{prop}}(m,n)]} \quad \text{(S13)}$$

As can be noticed in Eq.S13, the reconstructed phase distribution is obtained by a numerical evaluation of the arctan function, thus its values are restricted

in the interval $[-\pi, \pi]$, i.e., the phase distribution is wrapped into this range. To avoid possible ambiguities due to thickness differences greater than $\lambda/2$, phase-unwrapping methods have to be generally applied [7].

4 Intensity transmitted by a single valve for $\lambda = 532$ nm and $\lambda = 460$ nm

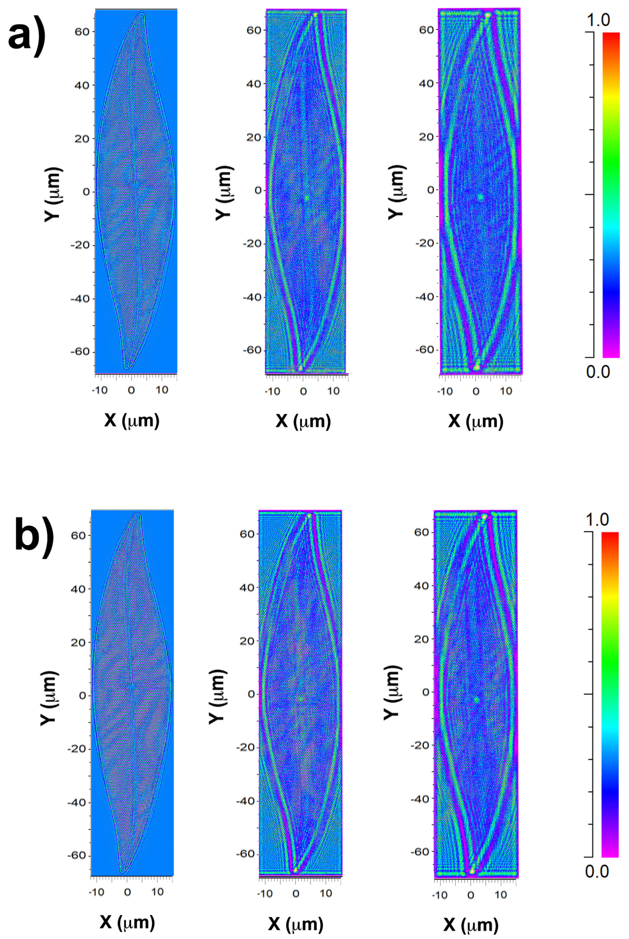

Figure S2: Color-encoded intensity transmitted by a single *P. strigosum* valve evaluated by WA-BPM for an incident wavelength of $\lambda = 532$ nm (**a**) and $\lambda = 460$ nm (**b**) at different distances along the optical axis $\hat{\mathbf{z}}$: immediately after the valve ($z = 0.4$ μm, first column); $z = 5$ μm (second column); $z = 10$ μm (third column). Incident intensity: 0.3 (a.u.). Fields propagating in air.

5 Transmitted intensity evaluated in XZ plane for different wavelengths

In Fig.S3 the spatial distribution of the intensity transmitted by a single *P. strigosum* valve when invested by a plane wavefront as numerically evaluated by WA-BPM is reported. The transmitted intensity has been calculated in the propagation plane XZ for different values of the incoming wavelength. Silica dispersion and absorption have been taken into account in performing the simulations. When passing from visible to UV-B radiation, the intensity of the transmitted radiation is progressively attenuated and radiation is spatially relocated farther from the valve.

6 UV-B extinction ratio for living cells in their aquatic environment

In Fig.S4 a transmission micrograph of a single live *P. strigosum* cell in its growth medium (enriched seawater F/2 medium) is shown when irradiated by UV-B ($\lambda = 280 - 315$ nm), together with two intensity profiles evaluated along a segment across a dark area (**a**) and an area including a brighter region (**b**), respectively. In both cases the transmitted intensity looks strongly attenuated, with $I_t/I_0 = 0.23 \pm 0.02$ along segment **a** and $I_t/I_0 = 0.64 \pm 0.02$ along segment **b** (I_t and I_0 standing for average transmitted and incident intensity, respectively).

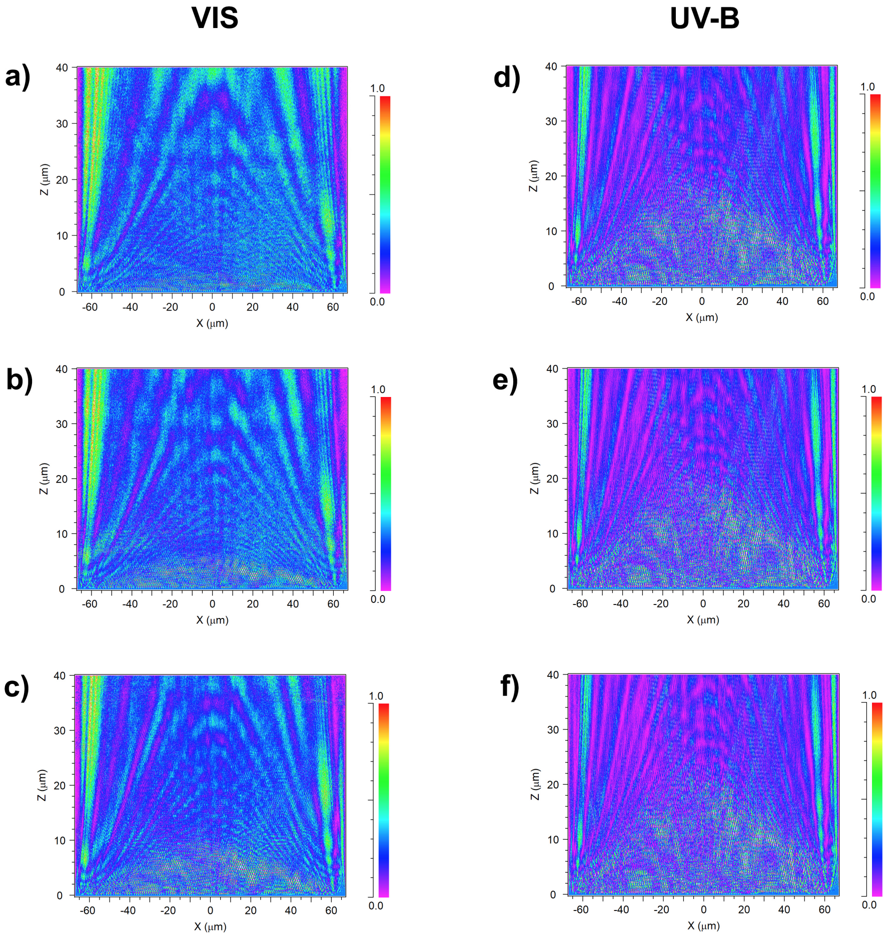

Figure S3: Numerically-evaluated, color-encoded intensity transmitted by a single *P. strigosum* valve when illuminated by a plane wave at different wavelengths. Radiation propagates along $\hat{\mathbf{z}}$ direction; *P. strigosum* valve lies orthogonally to the direction of propagation of the incoming wavefront and extends from $z = 0$ μm to $z = 0.4$ μm. The transmitted intensity has been evaluated in the XZ plane for $y = 0$ and up to $z = 40$ μm for the following incoming wavelengths: $\lambda = 633$ nm (**a**); $\lambda = 532$ nm (**b**); $\lambda = 460$ nm (**c**); $\lambda = 315$ nm (**d**); $\lambda = 300$ nm (**e**); and $\lambda = 280$ nm (**f**). Incident intensity: 0.3 (a.u.). Fields propagating in air.

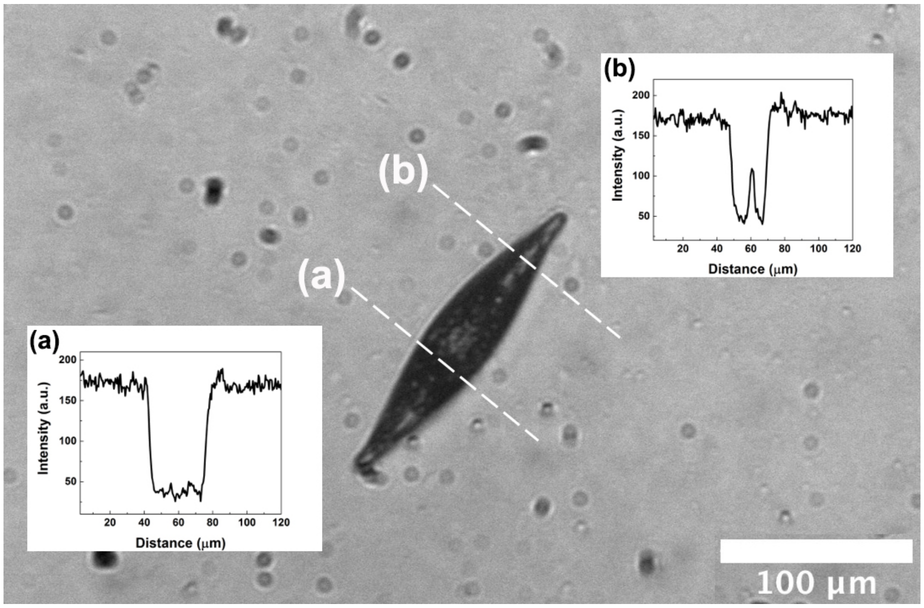

Figure S4: Intensity profiles evaluated along two different segments across a live *P. strigosum* cell in its growth medium (enriched seawater F/2 medium) when illuminated by UV-B radiation ($\lambda = 280 - 315$ nm): dark area (**a**); area including a brighter region (**b**).

References

[1] Hadley GR. Wide-angle beam propagation using Padé approximant operators. Optics Letters. 1992;17(20):1426-8.

[2] Hadley GR. Multistep method for wide-angle beam propagation. Optics Letters. 1992;17(24):1743-5.

[3] Ilic I, Scarmozzino R, Osgood RM. Investigation of the Padé approximant-based wide-angle beam propagation method for accurate modeling of waveguiding circuits. Journal of lightwave technology. 1996;14(12):2813-22.

[4] Yu L, Cai L. Iterative algorithm with a constraint condition for numerical reconstruction of a three-dimensional object from its hologram. JOSA A. 2001;18(5):1033-45.

[5] Goodman JW. Introduction to Fourier optics. Roberts and Company publishers; 2005.

[6] Nazarathy M, Shamir J. Fourier optics described by operator algebra. JOSA. 1980;70(2):150-9.

[7] Ferrara MA, Dardano P, De Stefano L, Rea I, Coppola G, Rendina I, et al. Optical properties of diatom nanostructured biosilica in Arachnoidiscus sp: micro-optics from mother nature. PLoS One. 2014;9(7):e103750.
